# A Comparative Evaluation of Third-Generation Advanced High-Strength Steels for Automotive Forming and Crash Applications

**DOI:** 10.3390/ma14174970

**Published:** 2021-08-31

**Authors:** Jacqueline Noder, Jon Edward Gutierrez, Amir Zhumagulov, James Dykeman, Hesham Ezzat, Clifford Butcher

**Affiliations:** 1 Department of Mechanical and Mechatronics Engineering, University of Waterloo, Waterloo, ON N2L 3G1, Canada; jegutierrez@uwaterloo.ca (J.E.G.); azhumagu@uwaterloo.ca (A.Z.); cbutcher@uwaterloo.ca (C.B.); 2 Honda R&D Americas Inc., 21001 State Route 739, Raymond, OH 43067, USA; dykemanjames7@gmail.com; 3 American Iron and Steel Institute, 2000 Town Center, Suite 320, Southfield, MI 48075, USA; hezzat@steel.org

**Keywords:** 3rd Gen AHSS, high-rate constitutive model, forming limit curve (FLC), Twist Compression Test (TCT), friction, formability prediction, Modified Maximum Force Criterion (MMFC), Marciniak, hole expansion, V-Bend, miniature dome

## Abstract

While the third generation of advanced high-strength steels (3rd Gen AHSS) have increasingly gained attention for automotive lightweighting, it remains unclear to what extent the developed methodologies for the conventional dual-phase (DP) steels are applicable to this new class of steels. The present paper provides a comprehensive study on the constitutive, formability, tribology, and fracture behavior of three commercial 3rd Gen AHSS with an ultimate strength level ranging from 980 to 1180 MPa which are contrasted with two DP steels of the same strength levels and the 590R AHSS. The hardening response to large strain levels was determined experimentally using tensile and shear tests and then evaluated in 3D simulations of tensile tests. In general, the strain rate sensitivity of the two 3rd Gen 1180 AHSS was significantly different as one grade exhibited larger transformation-induced behavior. The in-plane formability of the three 1180 MPa steels was similar but with a stark contrast in the local formability whereas the opposite trend was observed for the 3rd Gen 980 and the DP980 steel. The forming limit curves could be accurately predicted using the experimentally measured hardening behavior and the deterministic modified Bressan–Williams through-thickness shear model or the linearized Modified Maximum Force Criterion. The resistance to sliding of the three 3rd Gen AHSS in the Twist Compression Test revealed a comparable coefficient of friction to the 590R except for the electro-galvanized 3rd Gen 1180 V1. An efficient experimental approach to fracture characterization for AHSS was developed that exploits tool contact and bending to obtain fracture strains on the surface of the specimen by suppressing necking. Miniature conical hole expansion, biaxial punch tests, and the VDA 238-100 bend test were performed to construct stress-state dependent fracture loci for use in forming and crash simulations. It is demonstrated that, the 3rd Gen 1180 V2 can potentially replace the DP980 steel in terms of both the global and local formability.

## 1. Introduction

Stringent legislative requirements to reduce greenhouse gas emissions have fueled the development of advanced automotive sheet metals with superior mechanical properties to facilitate cold forming of complex geometries while providing sufficient strength levels for downgauging. With the intention to overcome the limited formability of the first generation of advanced high-strength steels (AHSS) and relatively high amount of alloying elements that can affect weldability of the second generation [1,2], the third generation of AHSS are being actively developed. The third generation of AHSS have become commercially available within recent years and are being actively considered by automakers due to their combination of high strength and superior ductility for structural safety components. The superior strength levels are obtained from a stress-state dependent phase transformation such that the retained austenite will transform into martensite and thus provide additional hardening to delay material instability during deformation. As reported by Chen et al. [3], the hardening response and resulting mechanical properties are dependent upon the initial volume fraction of retained austenite and the rate of the phase transformation. Davenport [4] reported that the formability of a 3rd Gen 980 steel can be comparable to a conventional DP590 steel, thus facilitating cost-efficient cold forming of complex A- and B-Pillars [5] while downsizing the sheet thickness for lightweighting purposes. Macek and Lutz [6] demonstrated stamping trials in which the in-production DP780 steel of a sill outer reinforcement could successfully be replaced with a thinner gauge of the 980 XG3^TM^ of US-Steel. Chen et al. [3] reported significantly improved formability across tensile stress states for a 3rd Gen QP980 steel from BAOSTEEL^®^ relative to a DP980. The FLC_0_ limit strain was 0.18 for the QP980 relative to 0.12 for the DP980 that can be attributed to the plasticity-induced phase transformation for the QP980 that stabilizes deformation by producing a higher work hardening rate and tensile elongation. Pednekar et al. [5] performed a structural performance comparison comprising three different crash scenarios and concluded that the 980 XG3^TM^ steel can potentially replace the PHS1500 steel using the same part geometry and gauge thickness while maintaining the crash performance. Pednekar et al. [5] emphasized the importance of the tribological conditions during forming despite approximations commonly adopted in forming simulations [7,8]. At present, the tribological data available for 3rd Gen AHSS is limited even though it can be critical for the virtual design of forming operations. 

In order to meet the targets of automakers for improved lightweighting and crash performance, the automotive steel industry has been developing optimized variants of the first generation of AHSS grades for specific applications, such as bending and flanging, while developing the third generation of steels in parallel. The proliferation of emerging steel grades has outpaced the ability of the steel producers and automakers to efficiently characterize and evaluate prototype grades. The past decade has seen tremendous improvements in the constitutive and fracture characterization of automotive sheet metals due to digital image correlation (DIC) and the adoption of hybrid experimental-numerical methods [9]. Although the hybrid approach is attractive, it is better suited for the characterization of mature grades when the automaker has decided upon a specific material for a component and can justify the time and cost for detailed model development. The hybrid-approach does not scale well to the needs of industrial users earlier in the development cycle as it requires a significant experimental campaign of test coupons followed by detailed finite-element analysis and inverse calibration by specialized analysts. The modelling choices and element mechanics induce subjectivity and thus only allow for limited comparisons between different testing labs. Although the fracture models may predict failure in the coupon tests near the target displacements, the fracture strains are numerical constructs that may not be realistic with respect to the measured fracture strains, especially when the inverse analysis was performed using shell elements. There is a clear need to develop efficient, experimentally-focused methodologies for constitutive, formability, and fracture characterization that facilitate the ranking of grades but are also amenable to industrial finite-element modelling of forming operations and crash events. It also remains an open question to what extent the characterization methodologies developed for conventional AHSS are applicable to 3rd Gen AHSS with their complex microstructure and stress-state dependent phase transformations. From an application perspective it remains unclear how consistent the mechanical properties are between different commercial 3rd Gen steel grades of the same nominal strength level given the variety of steels that fall under the current 3rd Gen designation. 

The motivation of the current paper is to provide a comprehensive analysis of the constitutive, formability, tribology and fracture behavior of three 3rd Gen AHSS which will be contrasted with conventional DP steels. To experimentally characterize the quasi-static constitutive response without inverse numerical analysis, a recently proposed methodology using tensile and shear tests was utilized as the shear tests are also required for fracture characterization. High-rate tensile tests at strain rates up to 1000 s^−1^ were also performed for the 3rd Gen AHSS and used to select and calibrate a rate-dependent constitutive model. To demonstrate the transferability of the experimental constitutive characterization to numerical applications, 3D tensile test simulations using solid elements were performed within LS-DYNA^®^. The predicted springback behavior of AHSS can be strongly affected by the plastic anisotropy [10] and evolving elastic properties [11]. To these ends, the materials were characterized for anisotropy and the degradation of the chord modulus in monotonic and sequentially-loaded tensile tests used to calibrate anisotropic yield functions and correlations for the chord moduli. The in-plane formability was characterized using Marciniak tests and it is shown that accurate, deterministic predictions of the FLC can be obtained using only the experimental constitutive data in the deterministic modified Bressan–Williams through-thickness shear model or the linearized Modified Maximum Force Criterion of Gutierrez et al. [12]. The tribological response was studied in Twist Compression Tests using the CommDraw220^®^ forming lubricant and steady-state friction coefficients for input in finite-element simulations are provided. Finally, an experimental approach was adopted to construct and calibrate the fracture loci. The proposed fracture tests can be readily performed in many test labs as the selected tests utilize punch contact and through-thickness strain gradients to suppress necking and obtain the fracture strains in proportional loading without numerical modelling. Recommendations for the tests and methods required to efficiently characterize and generate models for industrial modelling of AHSS are provided. 

## 2. Constitutive Characterization and Model Calibration

Three 3rd Gen AHSS with a nominal ultimate tensile strength level of 980 MPa, denoted as 3rd Gen 980, and 1180 MPa, denoted as 3rd Gen 1180 V1 and 3rd Gen 1180 V2, were selected along with two dual-phase steels of 980 MPa and 1180 MPa, and a 590R AHSS with a ferritic-martensitic microstructure. All steel grades were provided by the steel member companies of the Automotive Program of the American Iron and Steel Institute (AISI) (Southfield, MI, USA). AISI operates an anonymous steel sample bank in which members donate commercial grades for research projects. Since the project was composed of competing steel companies undertaking collaborative research, the identity of the supplier of respective steel grades, chemistry, and microstructural information was kept anonymous to all parties involved in the project, including the authors. It is emphasized that all steel grades on the project are commercially available. The three 3rd Gen steel grades and the 590R were supplied in a nominal sheet thickness of 1.4 mm and the DP980 steel had a sheet thickness of 1.2 mm. The DP1180 with a nominal sheet thickness of 1 mm was also provided by AISI and characterized using the same techniques by the University of Waterloo (Waterloo, ON, Canada). Details on the characterization of the DP1180 in plane strain bending, formability, and constitutive behavior have been reported in Noder et al. [13], Butcher et al. [14], and Abedini et al. [15]. Furthermore, the material dataset of the same lot of DP1180, which includes additional fracture data, is publicly available as part of the Numisheet 2022 fracture benchmark on non-linear strain paths [16].

### 2.1. Quasi-Static Hardening Response 

**Figure 1:**
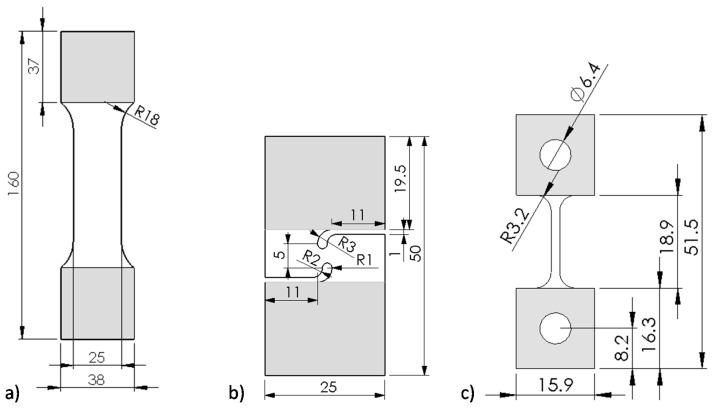
Geometries utilized to conduct tensile tests (**a**), simple shear tests [17] (**b**), and intermediate and high-rate tensile tests [18] (**c**). Note that all units are in mm and that the shaded area corresponds to the material section that was clamped during testing.

Tensile tests at an initial strain rate of 0.001 s^−1^ were performed utilizing a 100 kN electromechanical MTS Criterion 45 tensile frame (Eden Prairie, MN, USA). The JIS No. 5 geometry, as shown in Figure 1a, was extracted at 22.5° angles with respect to the sheet rolling direction (RD) and a minimum of three repeats were conducted for each test condition. Unless otherwise stated, full-field stereoscopic digital image correlation (DIC) technique and the software Vic3D-8^®^ of Correlated Solutions Inc. (Irmo, SC, USA), was adopted for optical strain measurements in all experiments. Settings related to DIC analysis along with the Virtual Strain Gauge Length (VSGL) are recorded in Table 1. 

**Figure 2:**
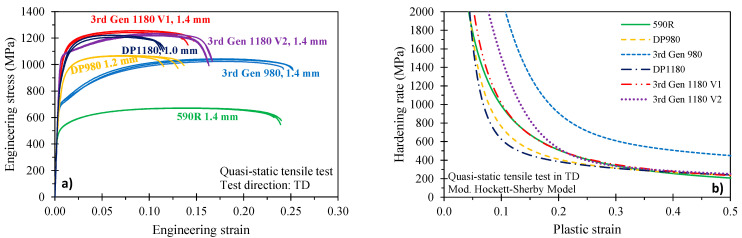
Engineering stress–strain response (**a**) and hardening rate obtained from the calibrated hardening model (**b**).

The engineering stress–strain curve and hardening rate of the studied steel grades are depicted in Figure 2a and the mechanical properties including the surface condition, yield strength (obtained from a 0.2% offset), ultimate tensile strength (UTS), yield-to-UTS ratio and uniform and total elongation are summarized in Table 2. Note that the uniform elongation was determined from the Considère criterion, which identifies diffuse necking at the intersection of the hardening rate with the true stress–strain curve. The elastic moduli of the undeformed material require alternate measurement techniques such as echo-pulse or resonant frequency analysis [11,19,20] since mechanical measurements are very sensitive to the measurement technique and associated with larger scatter [20]. Thus, the nominal value of 210 GPa, reported for DP980 steel [20] using resonant-frequency techniques, was adopted for this study. The ultimate strength level is comparable between the three 1180 MPa steel grades whereas the uniform elongation is 1.9% and 4.2% strain higher for the 3rd Gen 1180 V1 and V2, respectively. A similar trend is reflected in the total elongation. The uniform and total elongation of the DP980 and the 3rd Gen 980 is markedly different such that the strain for diffuse necking is more than twice as high for the 3rd Gen 980. The hardening rate was computed from the Modified Hockett-Sherby model (MHS) of Noder and Butcher [21] and is depicted for the sheet transverse direction (TD) in Figure 2b. For low strain levels of approximately 0.15 plastic strain, the hardening rate of the 3rd Gen 1180 V1 more resembles the behavior of the DP1180. The hardening response of the 3rd Gen 1180 V2 and the 3rd Gen 980, which feature more of a sigmoidal shape, is indicative of a higher amount of austenite transformation within the microstructure typical for 3rd Gen steels. The hardening rate of the DP980 resembles the observed behavior of the DP1180 steel.

**Figure 3:**
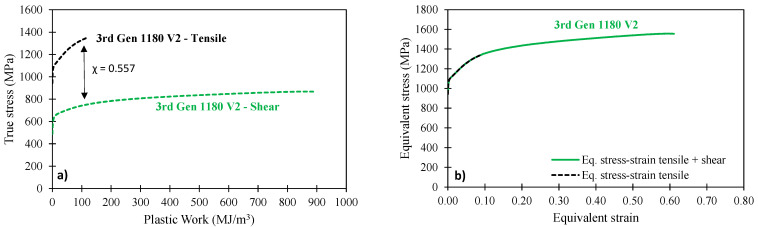
True stress versus plastic work (**a**) and converted equivalent stress-strain response for the 3rd Gen 1180 V2 (**b**).

Knowledge of the constitutive behavior to strain levels in excess of the diffuse necking strain in tensile tests is required to generate accurate predictions in automotive forming and crash simulations. Inverse numerical approaches have become increasingly adopted within industry and academia [22] but come at the cost of uncertainty surrounding the adoption of the plasticity model and element formulation [19]. Since shear tests are also required for fracture characterization of stress-state dependent fracture models such as the Modified Mohr–Coulomb (MMC) model [20,23], the shear test provides an efficient alternative to retrieve the material hardening response to significantly higher strain levels than in conventional tensile tests. In this study, the experimental plastic work-based methodology employed by Noder and Butcher [21] was adopted that utilizes the shear geometry of Peirs et al. [17], illustrated in Figure 1b. The shear tests were conducted with the transverse and rolling direction aligned with the principal tensile and compressive stress directions and the DIC settings summarized in Table 1 utilizing an AGX-V 50 kN Shimadzu tensile frame (Kyoto, Japan) and a crosshead speed of 0.003 mm/s. The plastic work in each test is computed as

(1) $$w_{pl}^{tensile}=\int \sigma_{1}d\varepsilon_{1}$$

(2) $$w_{pl}^{shear}=\int \tau d\gamma$$

where dγ is the incremental shear strain. The shear-to-tensile stress ratio, η, is identified at the plastic work at diffuse necking in the tensile test, as shown in Figure 3a. The tensile stress–strain response until the uniform elongation is retained and the converted shear hardening behavior is only adopted beyond the compromised tensile response since shear tests do not develop tensile localization. The incremental plastic work balance then allows computation of the equivalent stress–strain response utilizing

(3) $$\sigma_{eq}=\frac{\tau }{\eta }$$

(4) $$d\varepsilon_{eq}=\frac{dw_{pl}^{shear}}{\sigma_{eq}}$$

as visualized for the 3rd Gen 1180 V2 in Figure 3b. A detailed procedure of the shear conversion methodology is illustrated for the 3rd Gen 980 and 3rd Gen 1180 V1 in Gutierrez et al. [12].

Since the material frame rotates during simple shear deformation, the influence of shear anisotropy can be accounted for with additional shear tests and the methodology outlined in Abedini et al. [15]. The latter authors showed that for AHSS, the error in neglecting shear anisotropy was reported to be less than 2% and deemed to be sufficiently accurate for the purpose of this study. Once the experimental hardening data has been obtained, which is on the order of 60% or higher for AHSS, a phenomenological model can be calibrated. In the present study, the MHS model was calibrated in Matlab^®^ using a constrained calibration, *fmincon,* to ensure the predicted uniform elongation from the Considère criterion aligns with the experimental value. The MHS model is expressed such that

(5) $$\bar{\sigma }=G-\left(G-H\right)\exp\left(-I{\left(\varepsilon_{eq}^{}\right)}^{J}\right)+K\sqrt{\varepsilon_{eq}^{}}$$

where $\varepsilon_{eq}^{}$ corresponds to the equivalent plastic strain and *G-K* are calibration parameters identified in Table 3. Enforcing the Considère constraint upon calibration of the hardening model is paramount to avoid spurious behavior in the prediction of the forming limit curves, particularly in plane strain tension as demonstrated in Noder and Butcher [21]. The accuracy of the material response in 3D tensile simulations is discussed in Section 2.5.

### 2.2. Chord Modulus Evolution

**Figure 4:**
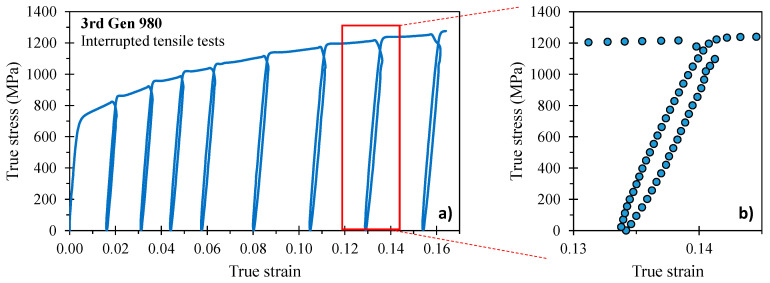
Interrupted tensile tests with a total of 8 unloading cycles for the 3rd Gen 980 (**a**) and close-up view of the non-linear unloading behavior, schematically shown for the seventh unloading cycle (**b**).

The reduction in the Young’s modulus with pre-strain, denoted as chord modulus, has been identified as the key parameter for accurate springback predictions [11,24]. Interrupted loading-unloading tensile tests under quasi-static conditions and utilizing the JIS No. 5 tensile geometry were performed in the AGX-V 50 kN Shimadzu tensile frame in the sheet transverse direction for all grades except the DP980 and DP1180. The specimen was loaded in displacement control at a test speed of 0.05 mm/s and unloaded at a rate of 800 N/s to maintain quasi-static deformation. The specimen was loaded and unloaded in 5–9 cycles to generate the stress-strain response schematically shown for the 3rd Gen 980 in Figure 4a.

The close-up view of the material unloading behavior, illustrated in Figure 4b, highlights the non-linear material response that has been reported to be caused by the presence of micro-plastic strain [24] due to (i) residual stresses owing to stress–strain partitioning and (ii) dislocation movement due to pile-up and repulsion [25,26]. Since there is no standardized procedure on how to measure the chord modulus, different approaches have been proposed in literature. Cleveland and Ghosh [24] computed the linear slope between the maximum and minimum load, which was noted by Govik et al. [27] to not necessarily correlate with the maximum strain prior to unloading and thus effect the magnitude of the chord modulus. Instead, Govik et al. [27] selected the two data points corresponding to the maximum and minimum strain whereas Sun and Wagoner [28] adopted the first data point after unloading for computation of the linear slope. To better capture the non-linear unloading behavior, Yoshida et al. [29] averaged the chord modulus over different stress ranges. The latter methodology was adopted for this study such that the chord modulus was computed from a line fit through the stress–strain response over a range of 0–95% of unloading. The empirical equation of Yoshida et al. [29] was calibrated using least squares optimization in Excel^®^ such that

(6) $$E_{chord}=E_{0}-\left(E_{0}-E_{S}\right)\left(1-\exp\left(-\xi \varepsilon_{eq}^{}\right)\right)$$

where $E_{0}$ refers to the Young’s modulus of the undeformed material, $E_{S}$ to the saturation modulus, and ξ represents a calibration parameter. The calibrated coefficients for Equation (6) are presented in Table 4. Note that within the calibration procedure, the initial guess for the undeformed Young’s modulus was set to the value of 210 GPa reported by Chen et al., [30] for DP980 steel using resonant frequency damping analysis. Unfortunately, there was insufficient material remaining for the DP980 and DP1180 to perform the chord modulus characterization tests. For completeness of the data set, the chord modulus data for DP980 and DP1180 provided in Table 4 was digitized from the study of Cobo et al. [31] and calibrated using Equation (6).

**Figure 5:**
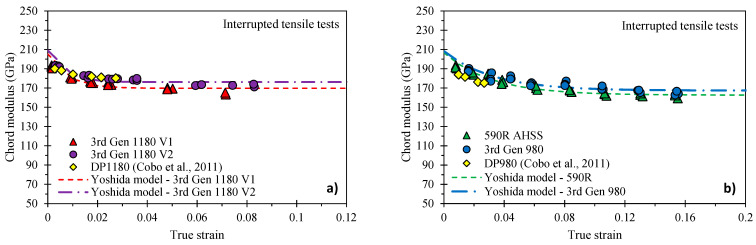
Measured and calibrated chord modulus evolution using the Yoshida model [29] for the 1180 MPa ultimate tensile strength steels (**a**) and the 590 MPa and 980 MPa strength steels (**b**). Note that the data for the DP980 and the DP1180 was digitized from the study of Cobo et al. [31].

Overall, the chord modulus reduction for all AHSS grades considered was similar with an average saturation modulus of 172 (±6.8) GPa. There does not appear to be a strong correlation between the strength level and chord modulus behavior although the 590R had the lowest modulus of approximately 163 GPa. The average saturation chord modulus compares well with the reported average of approximately 160 GPa for a total of 12 DP steels ranging from 500–980 MPa strength by Levy et al. [32] and a value ranging from 160–180 GPa for a series of DP980 steels of varying martensite volume fracture and hardness reported by Kupke [26]. The evolution of the chord moduli for the 1180 MPa steels are presented in Figure 5a with the 980 and 590R steels compared in Figure 5b.

### 2.3. Material Anisotropy

Sheet metals often experience a directionality in their mechanical properties induced during the rolling process which alters their crystallographic structure [33]. Therefore, the tensile anisotropy was studied in five different orientations in 22.5° increments with respect to the sheet rolling direction. Shear tests were conducted in one direction with the principal stresses aligned with the RD and TD of the sheet. The Lankford parameter was obtained by averaging the strain field of a rectangular 25 mm × 50 mm area over a range of strain that approximately corresponds to the uniform elongation (see Table 2 and Table 5). The stress ratios in Table 5 with respect to the stress in the RD correspond to the plastic work level in the limiting direction. Note that the anisotropy for the DP980 and DP1180 was studied in Abedini et al. [15] and the 3rd Gen 980 and 3rd Gen 1180 V1 in Gutierrrez et al. [12]. The normal anisotropy, $\bar{R}$, was computed from

(7) $$\bar{R}=\frac{1}{4}\left(R_{0}+2R_{45}+R_{90}\right)$$

where $R_{\theta }$ corresponds to the Lankford coefficient characterized at the angle θ relative to the sheet rolling direction.

**Figure 6:**
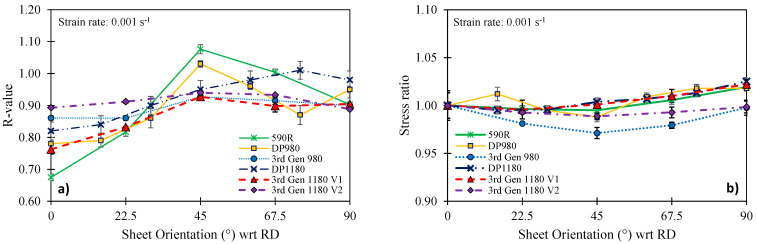
Comparison of R-value (**a**) and stress anisotropy (**b**) for the studied steel grades.

As depicted in Figure 6, the stress anisotropy of all studied steel grades was relatively low and the 3rd Gen steels are comparable to the observed behavior for DP steels. Overall, the strength in the transverse direction was about 2% higher than in the rolling direction. The normal anisotropy was close to unity (isotropy) and varied from 0.88 to 0.95 for the 3rd Gen 1180 V1 and the DP980, respectively, which highlights the mild plastic anisotropy in the studied steel grades. A similar trend is observed for the shear anisotropy with the 3rd Gen 1180 V2 being closer to a von Mises material, whereas the 3rd Gen 1180 V1 resembles the DP1180 with a shear stress ratio of greater than 0.6. The 3rd Gen 980 features slightly lower R-values than the DP980 and the 590R steel. The commercial 3rd Gen steels considered appear to have comparable or less anisotropy compared to conventional AHSS.

The phenomenological plane stress Barlat Yld2000-2d yield surface [34] was selected in this study. The yield criterion for Yld2000 is expressed as

(8) $$\sigma_{eq}^{Yld2000}={\left(\frac{{\left|X_{1}^{\prime }-X_{2}^{\prime }\right|}^{m}+{\left|2X_{2}^{\prime \prime }+X_{1}^{\prime \prime }\right|}^{m}+{\left|2X_{1}^{\prime \prime }+X_{2}^{\prime \prime }\right|}^{m}}{2}\right)}^{1/m}$$

in which $X_{1,2}^{\prime }$ and $X_{1,2}^{\prime \prime }$ are the principal values of the linearly transformed stress tensors $X_{ij}^{\prime }$ and $X_{ij}^{\prime \prime }$ which are related to the Cauchy stress through the fourth-order linear transformation tensors, $L_{ijkl}^{\prime }$ and $L_{ijkl}^{\prime \prime }$

(9) $$X_{ij}^{\prime }=L_{ijkl}^{\prime }:\sigma_{kl},\;X_{ij}^{\prime \prime }=L_{ijkl}^{\prime \prime }:\sigma_{kl}$$

which contain the anisotropy coefficients $\alpha_{1–8}$

(10) $$\left[\begin{array}{l} L_{11}^{\prime }\\ L_{12}^{\prime }\\ L_{21}^{\prime }\\ L_{22}^{\prime }\\ L_{66}^{\prime } \end{array}\right]=\left[\begin{array}{ccc} 2/3 & 0 & 0\\ -1/3 & 0 & 0\\ 0 & -1/3 & 0\\ 0 & 2/3 & 0\\ 0 & 0 & 1 \end{array}\right]\left[\begin{array}{l} \alpha_{1}\\ \alpha_{2}\\ \alpha_{7} \end{array}\right],\;\left[\begin{array}{l} L_{11}^{\prime \prime }\\ L_{12}^{\prime \prime }\\ L_{21}^{\prime \prime }\\ L_{22}^{\prime \prime }\\ L_{66}^{\prime \prime } \end{array}\right]=\left[\begin{array}{ccccc} -2 & 2 & 8 & -2 & 0\\ 1 & -4 & -4 & 4 & 0\\ 4 & -4 & -4 & 1 & 0\\ -2 & 8 & 2 & -2 & 0\\ 0 & 0 & 0 & 0 & 9 \end{array}\right]\left[\begin{array}{l} \alpha_{3}\\ \alpha_{4}\\ \alpha_{5}\\ \alpha_{6}\\ \alpha_{8} \end{array}\right]$$

**Figure 7:**
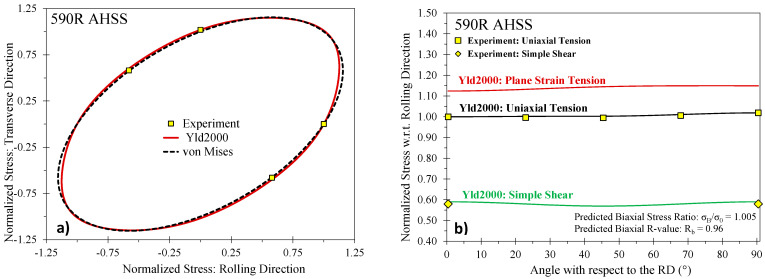
Calibrated Barlat Yld2000 yield surface for the 590R (**a**) and predicted stress ratios (**b**).

**Figure 8:**
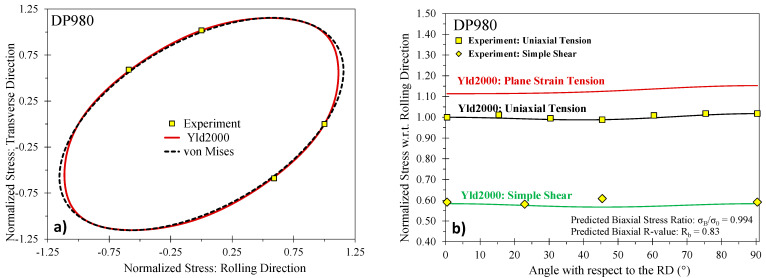
Calibrated Barlat Yld2000 yield surface for the DP980 (**a**) and predicted stress ratios (**b**).

**Figure 9:**
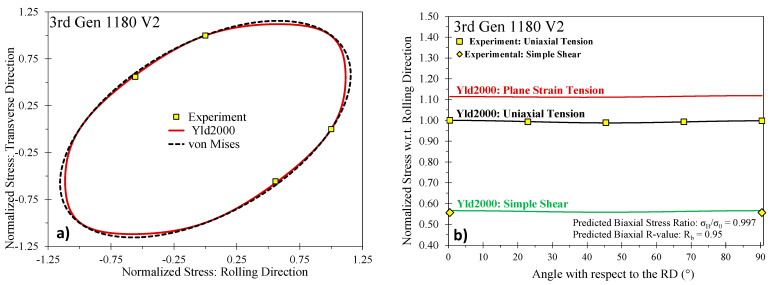
Calibrated Barlat Yld2000 yield surface for the 3rd Gen 1180 V2 (**a**) and predicted stress ratios (**b**).

The calibration parameters in Yld2000 do not directly correlate to the measured property data as with the Hill48 yield criterion where closed-form solutions exist. Numerical methods are required to identify the coefficients as discussed in Barlat et al. [34]. In the present study, the Yld2000 parameters were determined using least-squares minimization in Matlab^®^ with the experimental data in Table 5 as input. The calibrated yield surfaces are shown in Figure 7, Figure 8 and Figure 9 for the 590R, DP980, and 3rd Gen 1180 V2 with the calibrated anisotropy coefficients documented in Table 6. Note that the yield surface calibration for the 3rd Gen 980 and the 3rd Gen 1180 V1 was performed in Gutierrez et al. [12] and is included in Table 6 along with the data for DP1180 from Butcher et al. [14] for completeness. Note that for the 590R, DP980, and the 3rd Gen 1180 V2, no experimental data was available for the yield stress under plane strain tension and instead was predicted by the yield surface calibration to be 1.11 and 1.15 in the RD and TD for both the 590R and DP980, and 1.11 and 1.12 in the RD and TD for the 3rd Gen 1180 V2. Although not shown for brevity, the yield surfaces and anisotropy of the two 1180 MPa steels and the 3rd Gen 980 steel are comparable. The assumption of von Mises plasticity, commonly assumed in automotive crash simulations, would be a reasonable approximation for the 590R, DP980, DP1180 and the three 3rd Gen variants.

### 2.4. Dynamic Hardening Response

In addition to the quasi-static tensile tests discussed in Section 2.1, tensile tests in the TD were also performed at intermediate strain rates of 1 s^−1^ and 100 s^−1^ using the hydraulic intermediate strain rate (HISR) test frame developed by Bardelcik et al. [35]. Test speeds of 125 mm/s and 1250 mm/s were selected to obtain an average strain rate of approximately 1 s^−1^ and 100 s^−1^ using the miniature dogbone specimens of Smerd et al. [18] (see Figure 1c) with a gage length of 12.5 mm. 2D DIC was adopted using a Photron SA5 high speed camera to record images at frame rates summarized in Table 1.

**Figure 10:**
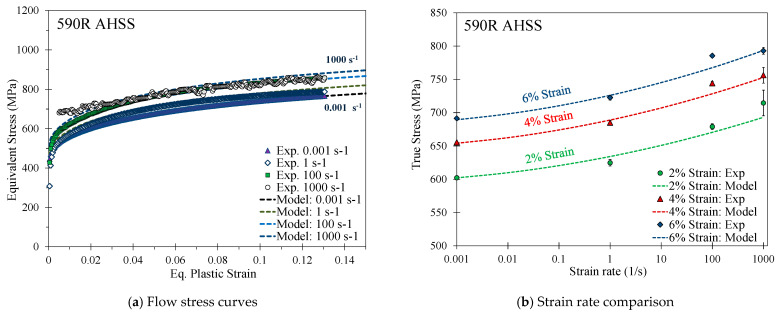
Calibrated quadratic Johnson Cook rate-dependent constitutive model for the 590R AHSS.

**Figure 11:**
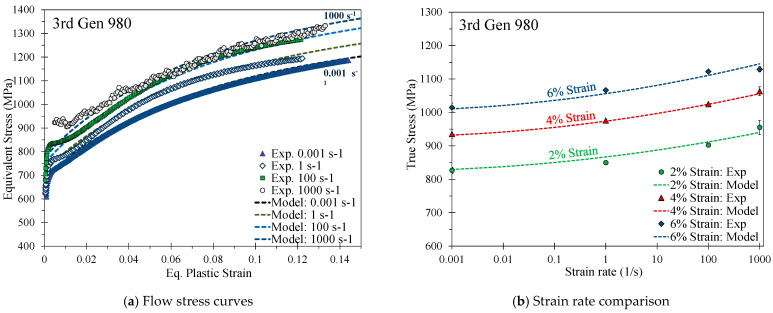
Calibrated quadratic Johnson-Cook rate-dependent constitutive model for the 3rd Gen 980.

**Figure 12:**
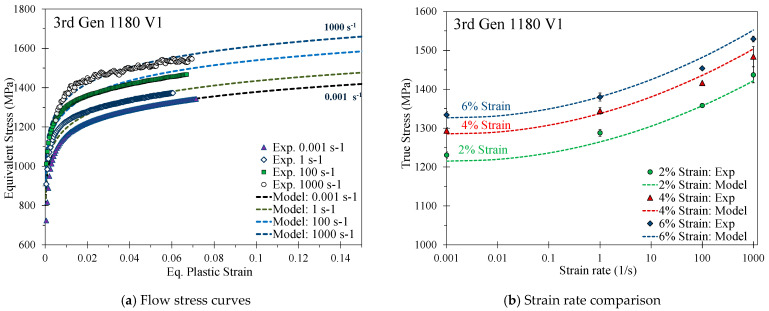
Calibrated quadratic Johnson-Cook rate-dependent constitutive model for the 3rd Gen 1180 V1.

Details on the THSB apparatus and testing methodology are provided in Rahmaan [36]. The measured strain rate effects for the 590R, 3rd Gen 980, and the 3rd Gen 1180 V1, shown in Figure 10, Figure 11 and Figure 12, were modeled using a modified Johnson-Cook model such that

(11) $$\bar{\sigma }\left(\dot{\varepsilon }\right)=\bar{\sigma }\left({\dot{\varepsilon }}_{ref}\right)\left[1+X\ln\left(\frac{\dot{\varepsilon }}{{\dot{\varepsilon }}_{ref}}\right)+Y{\left(\ln\left(\frac{\dot{\varepsilon }}{{\dot{\varepsilon }}_{ref}}\right)\right)}^{2}\right]$$

where $\bar{\sigma }$ corresponds to the flow stress at the quasi-static reference strain rate (${\dot{\varepsilon }}_{ref}$), and the coefficients *X* and *Y* are calibration parameters whose values are provided in Table 7.

**Figure 13:**
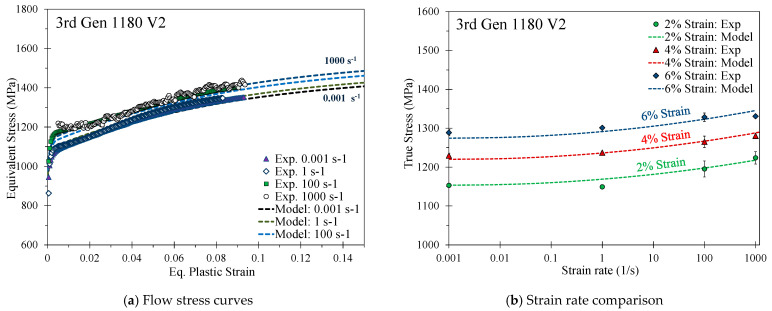
Calibrated modified Cowper Symonds rate-dependent constitutive model for the 3rd Gen 1180 V2.

For the 3rd Gen 1180 V2, which had lower strain rate effects as shown in Figure 13, the flow behavior was well-captured using a modified Cowper Symonds model to incorporate the logarithmic term as

(12) $$\bar{\sigma }\left(\dot{\varepsilon }\right)=\bar{\sigma }\left({\dot{\varepsilon }}_{ref}\right)\left(1+{\left[\frac{1}{C}\ln\left(\frac{\dot{\varepsilon }}{{\dot{\varepsilon }}_{ref}}\right)\right]}^{\frac{1}{p}}\right)$$

where *C* an *p* are calibration parameters summarized in Table 7.

Strain-rate effects for the DP980 steel were studied in Zhumagulov et al. [37] and calibrated using the modified Cowper–Symonds model in Equation (12) and the coefficients in Table 7.

**Figure 14:**
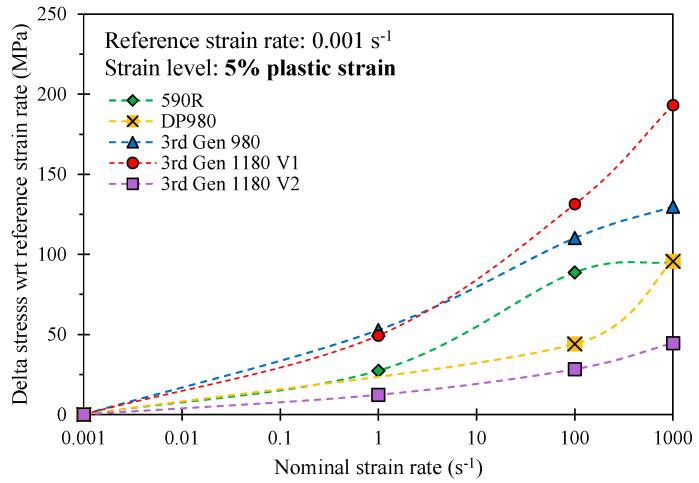
Comparison of the strain-rate induced increase in the flow stress at higher strain rates with respect to the stress level at the reference strain rate of 0.001 s^−1^ at a plastic strain of 5% for one representative repeat.

Figure 14 provides a comparison of the strain rate effects of one representative repeat utilizing the change in the stress at a respective strain rate with respect to the stress at the reference strain rate at a plastic strain level of 5%. In contrast to the 3rd Gen 1180 V1, the strain rate-sensitivity is rather mild for the 3rd Gen 1180 V2, particularly for the strain rates between 100–1000 s^−1^. The lower rate sensitivity of the 3rd Gen 1180 V2 is likely attributed to its stronger dependence upon microstructural transformation of austenite that is inhibited at higher strain rates and by higher local temperatures due to adiabatic heating [38]. A direct comparison of the rate-dependent stress increase reveals strain rate effects which are by a factor of approximately four greater for the 3rd Gen 1180 V1 (stress differential of 103 MPa and 149 MPa between a strain rate of 0.001–1000 s^−1^ for the 3rd Gen 1180 V2 and V1). No experimental rate-dependent data was available for the DP1180 Benchmark steel. The observed strain rate effects for the 3rd Gen 980 steel are slightly higher compared to the DP980 steel at a strain rate of 100 and 1000 s^−1^ and is more comparable to the 590R.

### 2.5. Evaluation of the Experimental Hardening Model in Tensile Simulations

To evaluate the experimental methodology of using the tensile and shear tests to obtain the hardening behavior, the accuracy of the calibrated rate-dependent constitutive model was assessed in 3D tensile simulations using an implicit integration scheme in the solver LS-DYNA^®^ (LSTC, Livermore, CA, USA). Owing to symmetry about two axes, only one quarter of the tensile specimen was simulated and discretized with fully-integrated solid elements of 0.3 × 0.3 × 0.14 mm^3^. A velocity of 0.05 mm/s was prescribed to the nodes on the top gripping area whereas the degree of freedom of the nodes in the bottom gripping area was fully constrained. The isotropic von Mises yield function was adopted for the 3D simulations since the Barlat Yld2000 model is limited to plane stress elements only and because the anisotropy of the AHSS was mild as discussed in Section 2.3.

**Figure 15:**
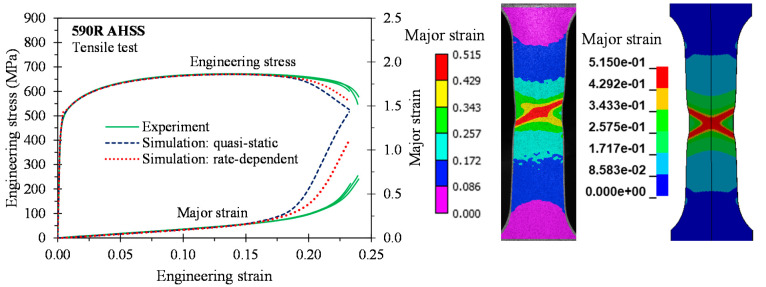
Comparison of the global and local response in 3D tensile simulations for the 590R.

**Figure 16:**
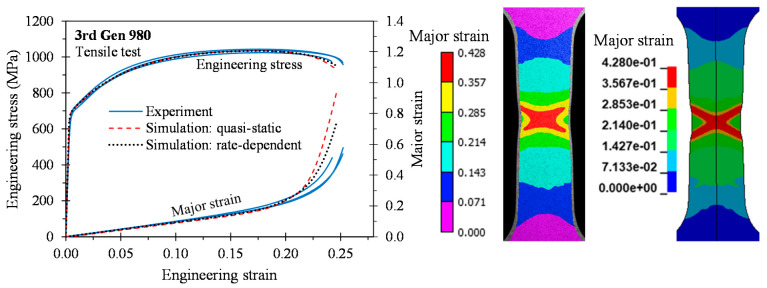
Comparison of the global and local response in 3D tensile simulations for the 3rd Gen 980.

**Figure 17:**
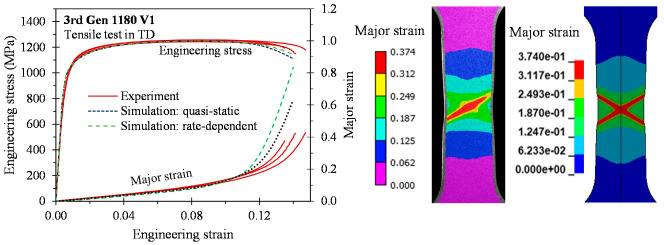
Comparison of the global and local response in 3D tensile simulations for the 3rd Gen 1180 V1.

**Figure 18:**
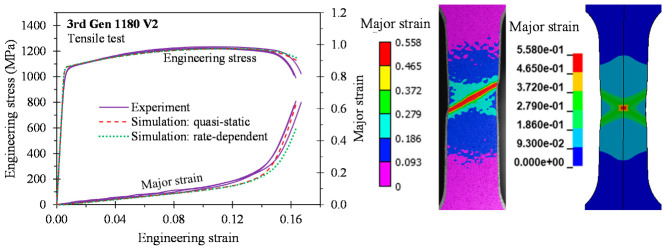
Comparison of the global and local response in 3D tensile simulations for the 3rd Gen 1180 V2.

As depicted in Figure 15, Figure 16, Figure 17 and Figure 18, the predicted global stress–strain responses are in good overall agreement for the 3rd Gen steels and the 590R. The predicted response for the 590R and 3rd Gen 1180 V1 is softer than observed in the tests which is attributed to the isotropic models predicting a severe double shear band for localization while only a single band formed in the tests. The predicted responses for the 3rd Gen 980 and the 3rd Gen 1180 V2 are in close agreement with the experiments. Limitations upon the mesh size in the finite-element model, isotropic assumption, and limited resolution for resolving local strains upon localization might be responsible for the observed deviation. The influence of the strain rate was relatively minor in the tensile tests such that both the quasi-static and rate-dependent constitutive model predict the local response to similar level of accuracy. Overall, the tensile simulations confirmed that the experimentally-derived constitutive model is sufficiently accurate for forming applications that will be demonstrated in the next section on analytical formability prediction.

## 3. Friction Characterization

**Figure 19:**
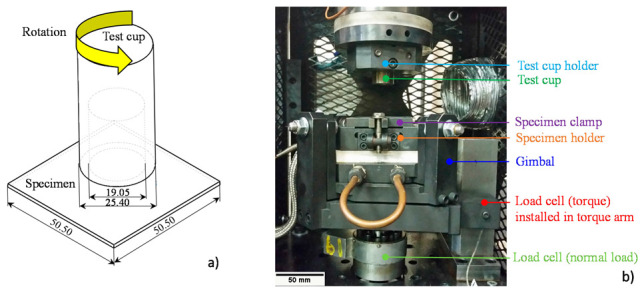
Schematic of the test methodology. Units are in mm. (**a**) and experimental set-up of the TCT. Units are in mm. (**b**).

Since frictional conditions affect the material flow behavior in a forming operation and thus affect the accuracy of model predictions, the tribological response of the 3rd Gen steel and the 590R at room temperature was studied in the Twist Compression Test (TCT) of Schey [39]. A recent study of Noder et al. [40] on aluminum alloys showed that good correlations between the TCT and forming operations could be established if the TCT process parameters were carefully selected. A schematic of the test methodology is depicted in Figure 19a.

The sheet specimen is pressed onto the test cup at a pressure determined by the user. Rotation is initiated at a predefined speed until the target sliding distance is reached. The isotropic coefficient of friction (*COF*) can be computed such that

(13) $$COF_{}=\frac{T}{r_{m}PA}$$

where *T* represents the measured reaction torque caused by the resistance to sliding, *A* the contact area, *r_m_* the mean cup radius and *P* the applied interface pressure. The test apparatus utilized in this study is depicted in Figure 19b. The test cup is positioned in the cup holder with the aid of a set screw and the specimen is located in the specimen holder. The angular rotation of the test cup is controlled through a hydraulic motor. The specimen holder is embedded in a gimbal assembly which serves as self-alignment mechanism to ensure parallel contact of the specimen and test cup during testing. The normal load is applied to the specimen through a hydraulic actuator with a 22.4 kN load cell installed. The frictional torque is measured with the aid of a 45 N-load cell positioned in the torque arm. For details on the test apparatus, the reader is referred to Noder et al. [40]. The process parameters of the friction test were carefully selected to represent an industrial forming process for AHSS using the CommDraw^TM^ 220 (Commonwealth Oil Corporation, Harrow, ON, Canada) lubricant provided by the automotive toolmaker, Bowman Precision Tooling. An interface pressure of 25 MPa, and a sliding distance of 75 mm were obtained from a forming simulation of a structural B-Pillar of the studied 3rd Gen 980 documented in Gutierrez et al. [41]. The sliding speed of 5 mm/s is on the lower bound of the forming speed in the B-pillar stamping process but was selected to produce a smooth and stable friction response. Square specimens were sheared to a length of 50.5 mm by 50.5 mm, deburred, and cleaned with acetone. The lubricant was applied with a pump spray to ensure even wetting of the specimen surface. A total of four repeats were conducted per test condition. Test cups made of Uddeholm Dievar^®^ (Uddeholm, Hagfors, Sweden) chromium-molybdenum-vanadium alloyed tool steel, hardened to 53 HRC (Rockwell hardness), were utilized for testing and replaced after each test.

**Figure 20:**
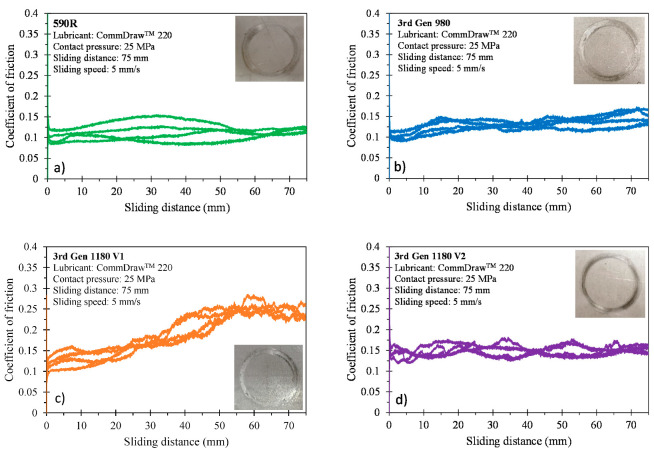
Evolution of the frictional response over the 75 mm sliding distance for the 590R (**a**), 3rd Gen 980 (**b**), 3rd Gen 1180 V1 (**c**) and 3rd Gen 1180 V2 (**d**).

The evolution of the frictional response during sliding is depicted in Figure 20. The initial COF was fairly similar for all four steel grades (COF of 0.1–0.16) with a slightly higher value for the 3rd Gen 1180 V2. The increase in the COF during sliding was most pronounced for the 3rd Gen 1180 V1 which reached a COF of approximately 0.25 at a sliding distance of 75 mm whereas the COF remained approximately constant for both the 590R and the 3rd Gen 1180 V2 and slightly increased for the 3rd Gen 980. Visual surface inspection post-test, embedded in Figure 20, revealed significant scoring for the 3rd Gen 1180 V1, some scoring for the 3rd Gen 980 and mild to negligible scratches for the 590R and the 3rd Gen 1180 V2. For input into a commercial finite-element solver, the complex nature of friction is often simplified to a single number. The ASTM G115 Standard guide for Measuring and Reporting Friction Coefficients [42] was followed to report a steady-state COF which excludes initial friction-induced spikes caused by initial contact. The steady-state COF is reported in Table 8. The ranking of the COF is in good agreement with visual inspection post-test: the highest values were found for the 3rd Gen 1180 V1 and the lowest for the 590R. Comparison of the surface roughness with the ranking of the COF provides only limited correlation since the 3rd Gen 1180 V1 with the highest frictional resistance had a comparable surface roughness to the 590R which featured the lowest COF. The TCT operates in the boundary lubricant regime which is governed by physical adsorption and adhesion/asperity interaction [43]. During physical adsorption, particles of atoms, ions, or molecules from the liquid phase reside on the metal surface to create a protective layer which reduces frictional effects during sliding [44]. These processes are governed by a change in the surface energy of the contacting partners and may have contributed to the higher COF observed in the 3rd Gen 1180 V1 which had an electro-galvanized coating.

## 4. Formability Characterization and Prediction

### 4.1. Marciniak Tests

**Figure 21:**
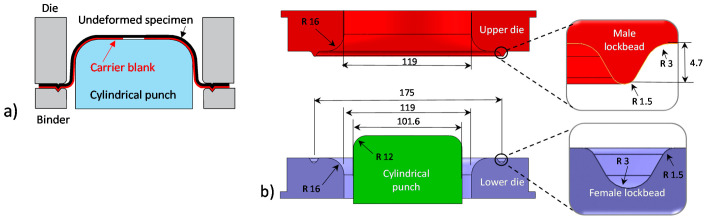
Schematic of methodology of Marciniak tests to characterize the limit strain under in-plane loading (**a**) and geometric dimensions of the tool set corresponding to the ISO12004-2 standard adapted from Gutierrez et al. [12]. All units are in mm (**b**).

In-plane forming limit curves (FLCs) were constructed in the transverse direction for all 3rd Gen steels and the 590R following the ISO12004-2 [45] standard for Marciniak tests as schematically shown in Figure 21a. To this end, an MTS formability press, equipped with a 600 kN loadcell and a 407 controlled was utilized. The formability characterization of the 590R and the 3rd Gen 1180 V2 was performed in the present study with the other grades characterized using the same toolset and procedures in Gutierrez et al. [12], Butcher et al. [14], and Noder and Butcher [21] for the other two 3rd Gen AHSS, DP1180, and DP980, respectively. The width of the dogbone specimens varied from 25.4 mm to 203.2 mm to explore stain paths ranging from uniaxial tension to equi-biaxial stretching. Die and punch dimensions corresponded to the ISO12004-2 [45] standard (see Figure 21b) and a lockbead with a 4.7 mm height was adopted to prevent material draw-in while clamping the specimen at a binder load of 640 kN. The punch ram speed was set to 0.25 mm/s and 0.1 mm thick circular Teflon layers lubricated with Vaseline were inserted between the sample and the carrier blank (washer) to promote fracture near the dome apex. The use of carrier blanks promotes in-plane stretching and localization within the center hole in the absence of tool contact stresses. For the draw side of the FLC, sandblasted 0.9 mm thick 1004 cold-rolled mild steel with a center hole of 32 mm was selected whereas 1.2 mm thick sandblasted stainless steel carrier blanks were selected for stretching-dominated strain path in light of higher ductility requirements. The sheet deformation was recorded using stereoscopic full-field DIC with the settings recorded in Table 1.

**Figure 22:**
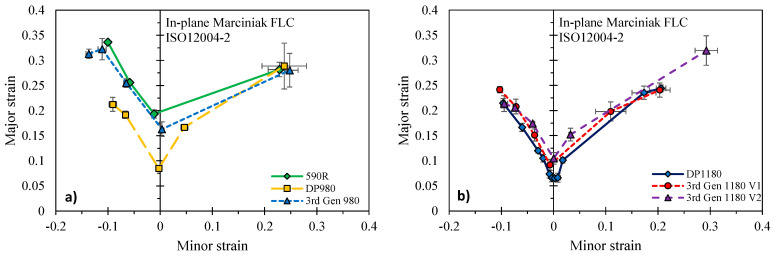
In-plane forming limit using the ISO12004-2 standard for the 590R, DP980, and 3rd Gen 980 (**a**) and the 3rd Gen 1180 V1, V2, and the DP1180 (**b**).

The ISO12004-2 method was adopted for identification of the forming limit strains in Figure 22 with the limit strains recorded in Table A1 and Table A2 in the Appendix A. In this method, the limit strain is determined from an inverse parabola fit through the strain distribution extracted from a line perpendicular to the crack in the last DIC image prior to fracture. The forming limit strain is determined from the corresponding maxima of the parabola. A comparison of the DP980 and the 3rd Gen 980 in Figure 22a highlights the superior global formability of the 3rd Gen 980 which is comparable to the limit strains obtained for the 590R. Particularly around plane strain tension, the most critical deformation mode for forming, the limit strain could be increased from approximately 9% to 16% strain when replacing the DP980 with the 3rd Gen 980. While the superior performance of the 3rd Gen steels is also reflected in the comparison with the 1180 MPa ultimate tensile strength steels in Figure 22b, the formability increase is more moderate around plane strain tension but superior under equi-biaxial stretching for the 3rd Gen 1180 V2.

### 4.2. Analytical FLC Prediction for In-Plane Stretching

From an experimental perspective, Nakazima tests are preferred due to their simplicity whereas only Marciniak tests are in agreement with the physical framework of most analytical FLC models in such that in-plane stretching (no bending) and a state of plane stress (no contact pressure) prevails. A series of empirical corrections to the Nakazima limit strains have been proposed by Min et al. [46]. It was demonstrated that the Nakazima limit strains are not as representative for in-plane stretching as commonly assumed. For these reasons, Marciniak tests were used in the present study as the limit strains obtained in the Marciniak tests do not require any corrections and can directly be utilized for comparison of analytical models.

The study of Gutierrez et al. [12] showed that both the linearized version of the Modified Maximum Force Criterion (MMFC) proposed in its original form by Hora et al. [47] and the extended through-thickness shear model proposed by Bressan and Williams [48] could more accurately predict the in-plane formability of the DP980, 3rd Gen 980, and the 3rd Gen 1180 V1 than the MK model of Marciniak and Kuzynski [49] and will be selected in this study. The MMFC [47] in Equation (14) is entirely deterministic and only requires the hardening model and yield surface as input. The localization process is idealized into two distinct stages of (i) uniform deformation until diffuse necking occurs, followed by (ii) a transition of the strain and stress path towards plane strain tension when an acute neck has formed.

(14) $$\begin{array}{cc} \mathrm{Homogeneous}\;\mathrm{Deformation} & \frac{\partial \sigma_{1}}{\partial \varepsilon_{1}}>\sigma_{1}\\ \mathrm{Onset}\;\mathrm{of}\;\mathrm{Diffuse}\;\mathrm{Neck}\;(\mathrm{Dorn}\;\mathrm{Model}) & \frac{\partial \sigma_{1}}{\partial \varepsilon_{1}}=\sigma_{1},\;\rho^{diff}=\rho =\frac{d\varepsilon_{2}}{d\varepsilon_{1}}\\ \mathrm{Diffuse}\;\mathrm{to}\;\mathrm{Acute}\;\mathrm{Necking}\;(\mathrm{MMFC}\;\mathrm{condition}) & \;\frac{\partial \sigma_{1}}{\partial \varepsilon_{1}}+\frac{\partial \sigma_{1}}{\partial \rho }=\sigma_{1},\;\rho \neq 0\\ \mathrm{Formation}\;\mathrm{of}\;\mathrm{Acute}\;\mathrm{Neck} & \rho =0 \end{array}$$

The diffuse necking criterion used within in the MMFC model has been misattributed to the Swift model [50] when it follows the model of Dorn and Thomsen [51] that applies the Considère criterion to stress states beyond uniaxial tension and only considers the major principal stress as critical instability metric. In uniaxial tension, the Considère, Dorn and Thomsen [51], and Swift [50] maximum force criteria are equivalent. Upon diffuse necking, the stress increment required to maintain neutral stability is governed by the second order plastic work rate. An acute has formed when a state of plane strain tension (ρ=0) is reached. The MMFC does not have a closed-form solution and requires a numerical integration procedure to satisfy the localization condition. The step-wise numerical procedure is provided in Hora et al. [47] and adopted in the present study. A stress-controlled integration loop in Matlab^®^ (MathWorks, Natick, MA, USA) was used to incrementally evaluate deformation until an acute neck was formed which defines the forming limit. For each stress state from uniaxial to biaxial tension, the plasticity model is incrementally integrated within the MMFC framework. Additional details related to the implementation with respect to the required partial derivatives are provided in Appendix B of the manuscript along with simplifications to von Mises plasticity. It is emphasized that the MMFC model of Hora et al. [47] is restricted to in-plane stretching and does not account for bending, tool contact stresses, or frictional effects. For consistency with the approximately linear strain path obtained in Marciniak tests, the strain path in the MMFC was linearized by adopting the strain ratio at diffuse necking, $\rho^{diff}$. The linearized strain components can readily be obtained using the incremental plastic work balance such that

(15) $$\varepsilon_{1}^{lin}=\frac{\varepsilon_{eq}}{k\left(\alpha \right)\left(1+\alpha \rho^{diff}\right)},\;k\left(\alpha \right)=\frac{\sigma_{1}}{\sigma_{eq}},\;\alpha =\frac{\sigma_{2}}{\sigma_{1}}$$

where α represents the stress ratio of the minor-to-major principal in-plane stress component and *k* to the ratio of the major to equivalent stress ($\sigma_{eq}$).

The modified Bressan–Williams model of Gutierrez et al. [12], termed BWx, provides a simple alternative to the incremental analysis of the MMFC model. Material instability for ρ≥0 occurs through the sheet thickness at the zero extension angle, *θ*, when the critical shear stress ($\tau_{cr}$) is reached. For ρ≤0 instability is determined from the constant maximum shear stress such that

(16) $$\tau_{cr}^{BWx}\left\{ \begin{array}{c} \frac{\sigma_{1}}{2}=\frac{k(\alpha )\bar{\sigma }\left(\varepsilon_{eq}\right)}{2},\;\;\;\;\;\;\rho \leq 0\\ \frac{\sigma_{1}}{2}\sin\left(2\theta \right)=k(\alpha )\bar{\sigma }\left(\varepsilon_{eq}\right)\frac{\sqrt{1+\rho }}{2+\rho },\;\rho \geq 0\; \end{array}\right.$$

The critical shear stress in the original model of Bressan and Williams [48] is treated a material constant to be calibrated from formability data. To remove the critical shear stress calibration, Gutierrez et al. [12] instead determined it from the Swift model [50] for diffuse necking under plane strain tension (ρ = 0). This condition requires determining the equivalent plastic strain according to

(17) $$k\left(\alpha \right)\frac{d\bar{\sigma }}{d\varepsilon_{eq}}=\bar{\sigma }\left(\varepsilon_{eq}\right)\left(\frac{1}{1+\alpha_{PS}}\right)$$

where $\alpha_{ps}$ is the minor-to-major principal stress ratio corresponding to the plane strain location for the yield surface. For von Mises, $\alpha_{ps}=0.5$. The critical shear stress can then be readily obtained from Equation (16). Implementation of the BWx model does not require numerical integration of partial derivatives for simple hardening models. A numerical solution is used in the present study due to the complexity involved with inverting the MHS hardening model.

**Figure 23:**
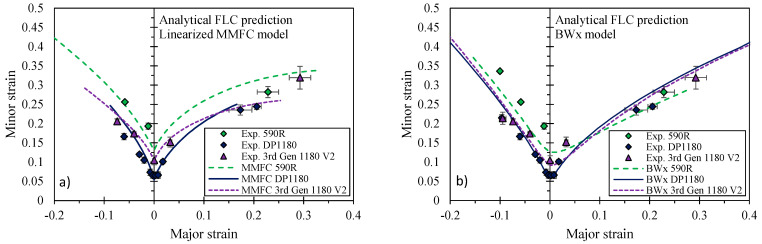
Formability prediction for in-plane stretching using the linearized MMFC (**a**) and the BWx model (**b**) for the 590R, DP1180, and the 3rd Gen 1180 V2.

As demonstrated in Figure 23, the analytical formability predictions are in very good agreement with the experimental limit strains. The linearized MMFC better captures the forming limits under drawing for the 590R whereas the BWx provides more accurate predictions for the biaxial limit strains. The BWx model does not consider the diffuse necking process and instead relies upon the critical shear stress which might be responsible for the underestimated limit strains on the draw side of the FLC that is associated with a significant diffuse necking process for more ductile steel grades. Contrary, on the stretch side, diffuse necking is usually minor and localization occurs through the sheet thickness which is accounted for in the BWx model. Although not shown for brevity, comparable levels of accuracy in the linearized MMFC and BWx models were also observed by Gutierrez et al. [12] for the DP980, 3rd Gen 980, and 3rd Gen 1180 V1. It is emphasized that accurate calibration of the hardening model to strain levels beyond the diffuse necking strain in a tensile test and reflecting the plastic uniform elongation are paramount for reliable and deterministic prediction of the forming limit. The forming limits of emerging grades of AHSS can be readily estimated from the analytical models using only the data from a tensile and shear test.

## 5. Fracture Characterization and Calibration of Fracture Locus

The local formability of AHSS and their performance within structural assemblies in a vehicle crash event is directly governed by the fracture behavior. The tensile and global properties are not sufficient to predict the local formability and fracture response. Although the tensile properties and global formability of the two 3rd Gen 1180 variants are similar, it will be shown in this section that the local response is markedly different. Conversely, the tensile and global formability of the DP980 and the 3rd Gen 980 are different while the local behavior is comparable.

**Figure 24:**
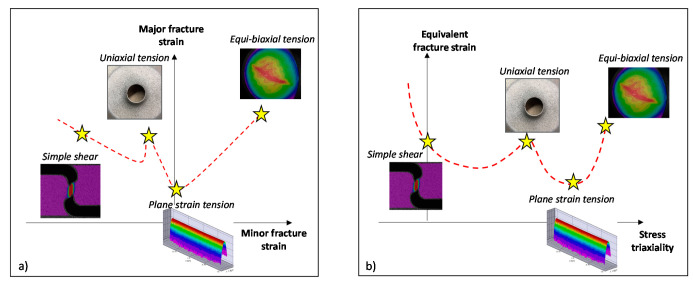
Overview of selected experimental program to characterize stress states ranging from simple shear to equi-biaxial tension, schematically illustrated in strain space (**a**) and stress space (**b**).

A purely experimental methodology for characterization and calibration of the fracture loci was adopted in this study and is schematically illustrated in Figure 24. The fracture characterization experiments were carefully selected by taking advantage of tool contact and bending mechanics to induce a through-thickness stress-strain gradient that delays and/or suppresses necking instability. Therefore, fracture initiation is shifted from the sheet mid-plane (sheet thickness) to the convex surface where strains can be directly measured with DIC. The primary advantage of this approach is that the experimental fracture strains can be directly measured in proportional loading without the complications of necking which alters the stress state. The equivalent failure strains reported in this section were obtained by integration of the strain path and adoption of the calibrated Barlat Yld2000 plasticity model, discussed in Section 2.3. The principal strains and von Mises effective strain assuming a linear strain path are recorded in the Appendix A in Table A3, Table A4, Table A5, Table A6, Table A7 and Table A8.

### 5.1. V-Bend—Plane Strain Fracture

**Figure 25:**
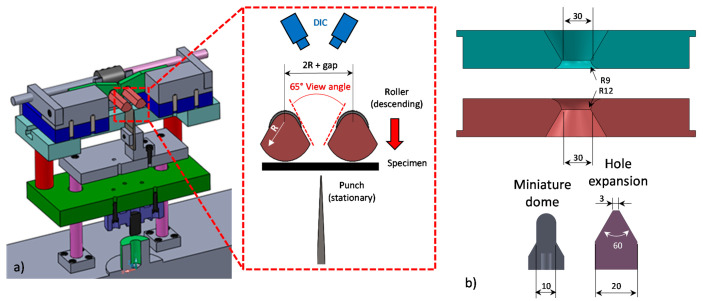
Schematic of the V-Bend frame utilized for characterizing the fracture strain under plane strain tension (**a**) and die set utilized to perform miniature dome tests and hole expansion tests to characterize the fracture limit under equi-biaxial tension and uniaxial tension. Units are in mm (**b**).

Accurate characterization of the plane strain fracture strain is vital since it represents the terminal strain state when an acute neck has formed and is the minima of the fracture locus. The VDA 238-100 [52] tight radius bend test provides an attractive alternative to conventional plane strain notch tests since fracture occurs on the specimen outer (convex) surface, where strains can directly be measured utilizing an inverted V-Bend frame equipped with a DIC set-up and thus eliminate the need for inverse finite-element analysis [53,54,55]. A schematic of the adopted test frame is depicted in Figure 25a. The set-up is inverted such that the punch is stationary and the rollers gradually descent to bend the specimen. The use of chamfered rollers provides a larger view angle to facilitate full-field stereoscopic DIC strain measurements of the entire bend width. The VDA 238-100 [52] recommendations were followed for selection of the process parameters (gap size of 2 times the sheet thickness + 0.5 mm, a 0.4 mm nominal punch tip radius, specimen dimensions of 60 mm × 60 mm and test speed of 0.33 mm/s). In light of the high ductility of the 590R steel, a punch with a nominal tip radius of 0.2 mm was selected. Details on the DIC analysis procedures for the V-Bend test are provided in Cheong et al. [54]. The VDA 238-100 specification recommends that failure be identified from a reduction in the punch load of 30 or 60 N (depending upon the material type). Noder et al. [53] highlighted limitations of this fracture detection methodology since materials with high bendability or thin gauges may not fracture in the tight-radius bend test and the punch force will decrease as a consequence of the kinematic boundary conditions of the test frame. A stress metric for fracture detection was proposed based upon the estimated bending moment, *M*. A reduction in the stress metric of 1% was found to provide results in agreement with the VDA force threshold for less ductile materials while it avoided false predictions of failure in ductile grades until a bend angle of 160 degrees. The stress metric is written as

(18) $$\Sigma =\frac{4}{t^{2}w_{}}M(F_{y},\alpha )=\frac{2F_{y}}{t^{2}w_{}}\left(\frac{t+0.25+R_{R}-\left(R_{R}+R_{P}\right)\sin\left(\frac{\alpha }{2}\right)}{\cos^{2}\left(\frac{\alpha }{2}\right)}\right)$$

with *w* as the initial specimen width and *t* the instantaneous sheet thickness which can be obtained from a phenomenological equation or from DIC measurements. The measured punch force is *F_y_*, whereas *R_R_* and *R_P_* are the respective radii of the rollers and punch with the bend angle, *α,* computed from the punch displacement using the relation in the VDA specification.

**Figure 26:**
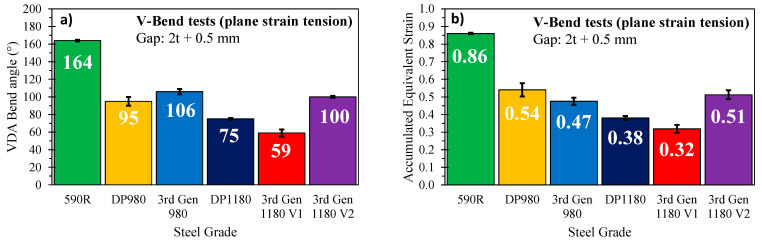
Failure bend angles (**a**) and equivalent strains (**b**) under plane strain tension. Note that the data for the plane strain fracture strain of the same lot of the DP1180 was reported in Noder et al. [13] for a von Mises material and was integrated using the Barlat Yld2000 plasticity model as part of this study.

The VDA bend angle and equivalent failure strains under plane strain tension depicted in Figure 26 highlight the superior performance of the 3rd Gen 1180 V2 grade with equivalent failure strains that are 0.19 and 0.13 greater than the 3rd Gen 1180 V1 and the DP1180, respectively. The 3rd Gen 1180 V1 can only withstand rather mild bending modes with a maximum bend angle of approximately 59° compared to a critical bend angle of about 100° for the 3rd Gen 1180 V2. The latter provides a much larger process window for forming parts with complex bend radii where through-thickness cracking under a plane strain mode is of primary concern. The plane strain tensile performance of the 3rd Gen 1180 V2 is comparable to the DP980 steel. The 3rd Gen 980 has a slightly lower failure strain under plane strain tension (0.07 equivalent strain) but provided forming limit strains which were twice as high for plane strain loading relative to the DP980. The failure strains for the 590R steel should be interpreted as a lower bound since the material performed a full bend in the tight-radius bend test with only minor hairline cracks.

### 5.2. Miniature Dome—Equi-Biaxial Fracture

**Figure 27:**
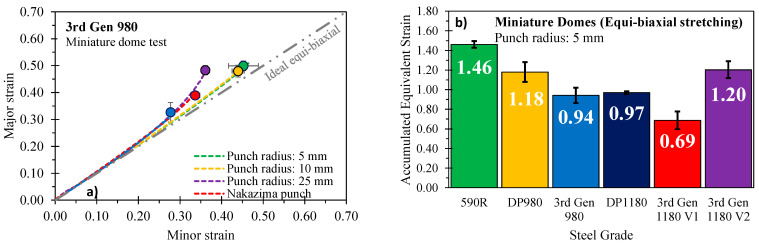
The effect of punch size on the linearity of strain path and the failure strain representatively shown for the 3rd Gen 980 (**a**) and integrated failure strains under equi-biaxial stretching (**b**). Note that the data for the DP980 was retrieved from Cheong [56] and the DP1180 from the Numisheet 2022 Benchmark [16] using the same test frame.

Miniature dome tests using a 5 mm punch radius were conducted utilizing the MTS formability press adopted for the formability characterization. The Marciniak die set was replaced with a miniature set-up depicted in Figure 25b and 120 grit Emery cloth was taped to the die and binder faces to prevent material draw-in during testing. Specimens were sheared to square blanks of 203 mm by 203 mm and deformed at a punch speed of 0.25 mm/s and a clamping load of 640 kN. Teflon film was adopted to promote failure without necking at the dome apex as illustrated for the 3rd Gen 980 in Figure 27a. Adoption of smaller punch radii induces a more severe stress-strain gradient through the sheet thickness that delays and/or suppresses tensile instabilities and thus provides higher failure strains under approximately proportional loading conditions [54]. Since failure is initiated on the convex surface of the test specimen, the failure strains could directly be extracted with a circular, 0.5 mm diameter, inspector tool in the software Vic3D^®^ of Correlated Solutions, Inc. The integrated failure strains in Figure 27b highlight the remarkable performance of the 3rd Gen 1180 V2 which clearly outperforms the two alternative 1180 MPa ultimate tensile strength steels, making it comparable to the DP980 steel. The equi-biaxial failure strains for the ductile 590R are still somewhat higher (by 0.26 equivalent strain) but come at the cost of half the strength level of the 3rd Gen 1180 V2 steel.

### 5.3. Shear Fracture

**Figure 28:**
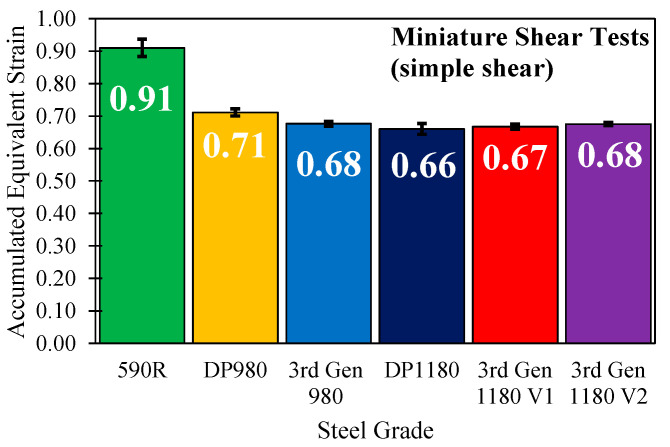
Integrated failure strains under simple shear loading. Note that the data for the DP1180 was retrieved from the Numisheet 2022 Benchmark [16].

The simple shear tests discussed in Section 2.1 have a twofold purpose in such that they can be utilized for constitutive characterization to obtain the hardening response to strains beyond tensile instability as well as for fracture characterization. Careful inspection of the shear tests for the 590R revealed that adoption of the geometry of Peirs et al. [17] resulted in edge fracture with rather low failure strains for the otherwise ductile 590R steel. Instead, an additional set of simple shear tests was performed utilizing the shear geometry of Roth and Mohr [57], who identified modified notch geometries for low, moderate, and high ductility alloys from an exhaustive numerical-experimental study. Adoption of the high-ductility specimen geometry for the 590R provided much higher failure strains and mitigated obvious edge cracking. The failure strains in Figure 28 were determined from the last image prior to fracture and the equivalent strain integrated using the finite strain solution of Butcher and Abedini [58] for simple shear deformation.

The failure strains under shear loading are similar with a maximum difference of 0.02 equivalent strain between the two 3rd Gen steel grades and the DP1180 steel. The same trend was observed for the DP980 and the 3rd Gen 980 steel with a difference in the equivalent strain of 0.03. The reduced effects under shear loading correlate well with reported literature data for TRIP (Transformation Induced Plasticity) steels which feature similar strength mechanisms. Jacques et al. [59], Shan et al. [60], and Blondé et al. [61] reported an explicit dependence of the stress state upon the austenite transformation rate with lowest amounts of transformed austenite for shear modes and increased amounts for deformation modes with higher triaxiality such as uniaxial tension and equi-biaxial tension. Although obvious edge cracking was not observed in the tests it appears likely that edge fracture is responsible for the similar values in the five steel grades.

### 5.4. Conical Hole Expansion with a Machined Edge—Uniaxial Fracture

**Figure 29:**
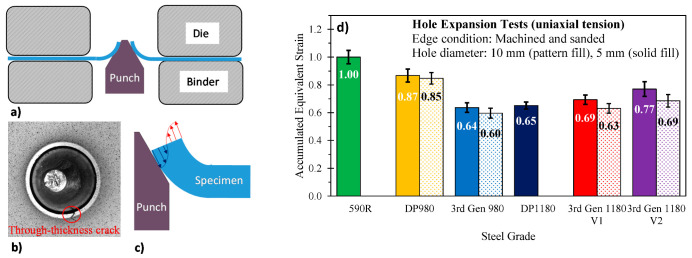
Schematic of hole expansion test (**a**), fracture initiation through the presence of a through-thickness crack for the 3rd Gen 1180 V1 (10 mm initial hole diameter) (**b**), schematic of the through-thickness strain gradient to suppress necking (**c**), and effect of the initial hole size (5 mm versus 10 mm diameter) for different steel grades (**d**). Note that the data for the DP1180 was retrieved from the Numisheet 2022 Benchmark [16] and the DP980 (10 mm hole size from Cheong [56], with both materials tested on the same equipment.

Conical hole expansion tests are commonly utilized for sheared edge formability studies. As schematically illustrated in Figure 29a, the test consists of expanding a central hole in a clamped sheet specimen using a concial punch with an inclined angle of 60° until a through-thickness crack forms at the hole edge as shown for the 3rd Gen 1180 V1 in Figure 29b. Fracture is initiated on the outer hole diameter whereas the through-thickness stress gradient from compression (punch contact) to uniaxial tension in circumferential stretching (outer edge) suppresses tensile instabilities (see Figure 29c). The mechanics of conical hole expansion tests are particularly well suited for uniaxial fracture characterization. A machined hole edge condition was used in the present work to obtain the uniaxial fracture limit of the material without complicating effects of a severely work-hardened sheared edge using the methodology of Pathak et al. [62]. The principal and equivalent strains in the conical punch can be obtained from the outer diameter at fracture and the tensile R-value in the direction perpendicular to the crack location, defined as $R_{\theta +90{}^{\circ}}$, as

(19) $$\varepsilon_{eq}^{}\approx \varepsilon_{1}^{}=\ln\left(\frac{d_{outer}^{f}}{d_{o}}\right)$$

(20) $$\varepsilon_{2}^{}=\frac{-\varepsilon_{1}^{}}{\left(1+\frac{1}{R_{\theta +90{}^{\circ}}}\right)}$$

where $d_{outer}^{f}$ corresponds to the measured outer diameter at failure and $d_{o}$ to the initial, undeformed, hole diameter. The MTS formability tester and the die set utilized for the miniature dome tests in Figure 25b was also adopted for the hole expansion tests but the hemispherical punch was replaced with a conical punch, also illustrated in Figure 25b. The hole expansion test specimens consisted of square 203 mm × 203 mm blanks with a central hole drilled, reamed, and then sanded with SiC paper to remove burrs and minimize the potential for premature fracture due to surface defects caused by machining. To investigate the influence of the hole diameter, both 5 mm and 10 mm hole diameters were considered. The test specimens were then clamped at 640 kN and expanded with the conical punch at a velocity of 0.25 mm/s while the stereo DIC camera system recorded the test. Since DIC cannot measure the strain at an edge, the software Image-Pro Plus^®^ (Media Cybernetics, Rockville, MD, USA) was adopted to measure the inner and outer diameter of the expanded hole in the image when a through-thickness crack was detected. The effect of the initial hole size upon the equivalent failure strain is visualized in Figure 29d. For all studied steels, the smaller hole size provided slightly higher fracture strains since the smaller initial hole size led to an increase in the strain gradient around the hole and thus delayed acute localization. These observations are consistent with the findings of Hino et al. [63] who reported higher failure strains in hole expansion tests of 10 mm center holes by changing the inclined angle of the punch to vary the imposed strain gradient.

The studies of Levy and van Tyne [64] and Pathak et al. [62] established correlations between the hardening rate of steels and the edge formability. It was found that a high hardening rate is unfavorable in edge formability owing to a high stress differential between phases which initiates void formation at lower macro strains. This conclusion is in line with the superior edge formability for the DP980 which had a lower hardening rate (see Figure 2b) relative to the 3rd Gen 980. Contrary, the DP1180 and the two 3rd Gen 1180 MPa strength steels do not seem to correlate with this trend since the 3rd Gen 1180 V2 featured approximately similar or slightly higher failure strains relative to the DP1180 despite a higher hardening rate at uniform elongation. Among steel grades, the 3rd Gen steels can be seen in analogy to TRIP steels with their complex microstructure which enables a stress-state dependent phase transformation to provide additional hardening during deformation. Interestingly, Levy and van Tyne [64] commented on some differences between the material performances of TRIP steels owing to the complex microstructure and variation in the production methods. A similar or slightly lower performance in hole expansion tests of TRIP steels relative to conventional steels was reported in the study of Konieczny and Henderson [65].

### 5.5. Comparison of Global and Local Formability

**Figure 30:**
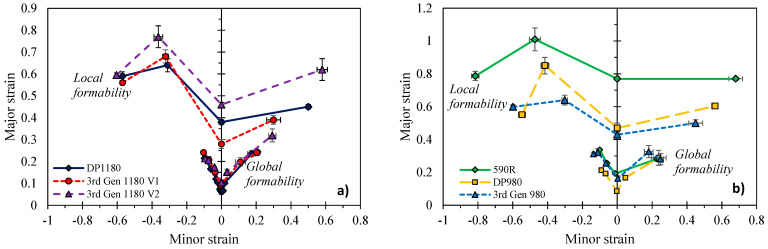
Comparison of the local and global formability in strain space for the 1180 MPa ultimate tensile strength (**a**) and the 590 MPa and 980 MPa strength steels (**b**).

The failure strains obtained under different loading conditions are summarized in Figure 30 and compared to the global formability discussed in Section 4. Overall, the DP1180 and 3rd Gen 1180 V1 are expected to behave in a similar manner during forming and crash operations as the 3rd Gen 1180 had marginally better global formability but slightly lower local formability than the DP steel. The similarities between these two grades are not unexpected based upon the similar hardening behavior where the 3rd Gen 1180 V1 did not show obvious signs of austenite transformation. In contrast, the 3rd Gen 1180 V2, which showed significant transformation effects in the hardening response, exemplifies the potential of third generation steels. The global formability of the two 3rd Gen 1180 steels was similar while the significantly higher local formability of the 3rd Gen 1180 V2, particularly in biaxial modes, makes it an attractive candidate for automotive structural applications. The lower strain rate sensitivity of the 3rd Gen 1180 V2 will also help reduce press tonnage compared to the V1 which exhibited an increase three times higher.

**Figure 31:**
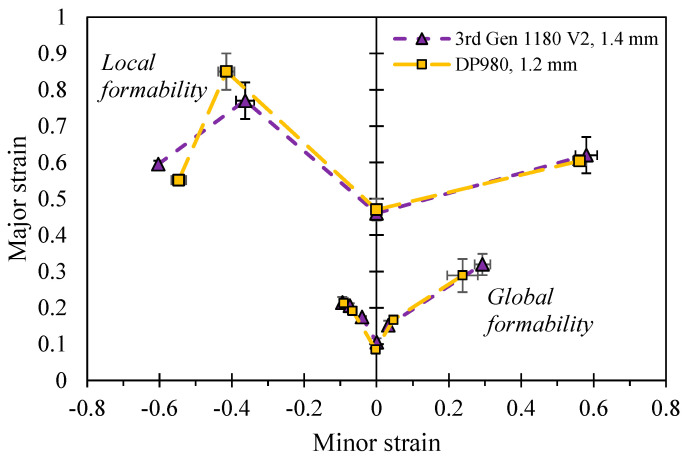
Comparison of the local and global formability in strain space for the 3rd Gen 1180 V2 and the DP980.

The opposite trend was observed for the 3rd Gen 980 steel which featured superior global formability over the DP980 and thus provides appealing mechanical properties for cold forming of complex part geometries whereas the local formability is comparable or lower than the conventional DP980 steel. It is expected that more complex geometries will be feasible for the 3rd Gen 980. However, without increased local fracture limits and a larger part of its ductility consumed by a more complex forming process, its subsequent performance in a crash event remains unclear and is a topic for future work. A direct comparison between the global and local performance of the 3rd Gen 1180 V2 and the DP980 in Figure 31 demonstrates that except for fracture under a stress state of uniaxial tension, the 3rd Gen 1180 V2 has the potential to replace the DP980 steel. The present study clearly highlights the variation in mechanical properties within the current class of commercial 3rd Gen steels and provides the experimental methodologies for their characterization to aid in material selection and numerical design.

### 5.6. Calibration of Fracture Locus

Since the experimental campaign for fracture characterization was carefully selected to avoid strongly non-proportional loading and fracture initiation from the sheet mid-plane where DIC cannot be utilized, the fracture model selected for this study can directly be calibrated with the equivalent failure strains as a function of the stress triaxiality. Rahmaan et al. [66] proposed the isotropic Generalized Drucker–Prager (GDP) fracture model which can be utilized in an equivalent strain framework adopting the integrated equivalent failure strains obtained in the experiments. The equivalent von Mises stress in the Drucker–Prager [67] criterion is replaced with the non-quadratic Hosford [68] model and the weighting of the intermediate stresses is controlled through the material parameters c and b whereas β corresponds to a critical stress value for when fracture occurs

(21) $$\sigma_{eq}^{Hosford}+c\left(\sigma_{1}+\sigma_{3}\right)+b\sigma_{2}=\beta ,\;\sigma_{eq}^{failure}\left(T,{\bar{\theta }}_{L}\right)=\frac{\beta }{g_{GDP}\left[\sigma_{ij}/\sigma_{eq}^{vM}\right]}$$

(22) $$\sigma_{eq}^{Hosford}={\left(\frac{1}{2}\left[{\left|\sigma_{1}-\sigma_{2}\right|}^{a}+{\left|\sigma_{2}-\sigma_{3}\right|}^{a}+{\left|\sigma_{3}-\sigma_{1}\right|}^{a}\right]\right)}^{\frac{1}{a}}$$

where a corresponds to the Hosford exponent, T is the stress triaxiality defined in terms of the ratio of the hydrostatic stress to the equivalent stress, and $\bar{\theta }$ is the dimensionless Lode angle parameter. Adoption of the Haigh–Westergaard coordinate system provides a functional form of Generalized Drucker–Prager (GDP) function

(23) $$\begin{array}{cc} g_{GDP}\left[\sigma_{ij}/\sigma_{eq}^{von\;Mises}\right] & ={\left(\frac{1}{2}\left({\left|f_{1}-f_{2}\right|}^{a}+{\left|f_{2}-f_{3}\right|}^{a}+{\left|f_{1}-f_{3}\right|}^{a}\right)\right)}^{\frac{1}{a}}\\ & +c\left(2T+f_{1}+f_{3}\right)+b\left(T+f_{2}\right) \end{array}$$

(24) $$f_{1}=\frac{2}{3}\cos\left[\frac{\pi }{6}\left(1-{\bar{\theta }}_{L}\right)\right],\;f_{2}=\frac{2}{3}\cos\left[\frac{\pi }{6}\left(3+{\bar{\theta }}_{L}\right)\right],\;f_{3}=-\frac{2}{3}\cos\left[\frac{\pi }{6}\left(1+{\bar{\theta }}_{L}\right)\right]$$

The dimensionless Lode parameter is expressed in terms of the third deviatoric stress invariant $\left(J_{3}\right)$ and the equivalent von Mises stress $\left(\sigma_{eq}^{vM}\right)$

(25) $${\bar{\theta }}_{L}=1-\frac{2}{\pi }\cos^{-1}\left(\frac{27}{2}\frac{J_{3}}{{\left[\sigma_{eq}^{vM}\right]}^{3}}\right)$$

(26) $$J_{3}=-\frac{1}{27}\left(\sigma_{1}+\sigma_{2}-2\sigma_{3}\right)\left(\sigma_{2}+\sigma_{3}-2\sigma_{1}\right)\left(\sigma_{1}+\sigma_{3}-2\sigma_{2}\right)$$

(27) $$\sigma_{eq}^{vM}=\sqrt{\sigma_{1}^{2}+\sigma_{2}^{2}+\sigma_{3}^{2}-\left(\sigma_{1}^{}\sigma_{2}^{}+\sigma_{1}^{}\sigma_{3}^{}+\sigma_{2}^{}\sigma_{3}^{}\right)}$$

**Figure 32:**
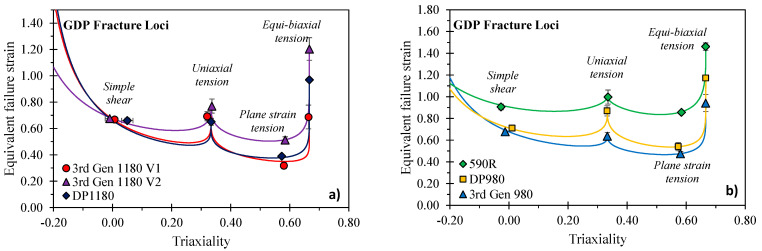
Calibrated fracture *loci* using the Generalized Drucker–Prager fracture model of Rahmaan et al. [66] for the 1180 MPa strength steels (**a**) and the 590 MPa and 980 MPa strength steels (**b**).

The weighting factors introduced in Equation (21) mitigate the limitation of equal equivalent fracture strains in uniaxial tension and equi-biaxial stretching encountered in the Hosford–Coulomb (HC) model of Mohr and Marcadet [69]. The Hosford–Coulomb model is recovered for b=0, the Drucker–Prager model for a=2 and b=c, and the Mohr–Coulomb model with a=1 and b=0. For simplicity, the GDP model can be converted into an equivalent strain-based form for that Equation (21) is rewritten to

(28) $$\varepsilon_{eq}^{failure}=\frac{\beta^{\prime }}{g_{GDP}\left[\sigma_{ij}/\sigma_{eq}^{von\;Mises}\right]}$$

where $\beta^{\prime }$ corresponds to a critical strain value utilized as a calibration parameter. Rahmaan et al. [66] identified the four calibration parameters (a, b, c and β) utilizing non-linear best fitting whereas in this study, a deterministic approach is adopted instead. Closed-form solutions for the parameters b, c and β in dependence of the Hosford exponent a, can be derived when considering the stress states of simple shear (SS), uniaxial tension (UT), plane strain tension (PST), and equi-biaxial tension (EBT). The Hosford exponent can then be obtained by adoption of the Newton–Raphson method. Details on the implementation and the objective function are documented in Appendix C. The calibrated fracture *loci* are depicted in Figure 32 with the calibration coefficients documented in Table 9. The plane strain valley typically represents the minima of a fracture locus and is the most critical loading condition, particularly for bending-dominated forming operations and folding behavior for energy absorption in crash. The local formability of the 3rd Gen 1180 V2 is superior to the 3rd Gen 1180 V1 despite the similar global formability discussed in Section 4. The fracture loci of the 3rd Gen 980 are similar to the DP980 and markedly lower than the 590R but comes with a superior global formability relative to the DP980.

## 6. Conclusions

A comprehensive experimental study on three 3rd Gen AHSS with a nominal ultimate tensile strength of 980 and 1180 MPa, one DP980 steel, and one 590R steel grade was performed and contrasted with the Numisheet 2022 data compiled by the authors for the DP1180. The following conclusions can be drawn:

- The anisotropy of all three studied 3rd Gen steels is comparable to conventional AHSS such as DP steels. The rate-sensitivity was found to significantly vary between the two 3rd Gen 1180 steels and is attributed to the differential in microstructure transformation.
- The chord modulus reduction with pre-strain was similar for all studied steel grades (saturation chord modulus of approximately 162–176 GPa) and correlated well with literature data for the DP980 (175 GPa) and DP1180 (182 GPa).
- The frictional response in the presence of the CommDraw^TM^ 220 lubricant was similar for the 590R, 3rd Gen 980, and 3rd Gen 1180 V2 whereas the electro-galvanized 3rd Gen 1180 V1 steel experienced higher resistance to sliding with an average friction coefficient of 0.19.
- The in-plane formability characterized using Marcinak tests was accurately predicted utilizing the deterministic linearized MMFC and the BWx model that only required the hardening model and calibrated yield surface as input parameters. Both 3rd Gen variants featured a slightly higher FLC_0_ limit strain (0.07–0.10) compared to the conventional DP1180 steel (0.05) and a marked improvement from approximately 0.07 to 0.16 for the 3rd Gen 980 relative to the DP980.
- The superior local formability of the 3rd Gen 1180 V2 relative to the conventional DP1180 steel was limited to stress states of equi-biaxial, plane strain, and uniaxial tensile loading. Overall, the DP1180 and 3rd Gen 1180 V1 were comparable in terms of local and global formability performance while the 3rd Gen 1180 V2 demonstrated the potential of 3rd Gen steels to provide significant gains in the fracture response and a moderate increase in formability. The 3rd Gen 1180 V2 has the potential to replace the DP980 in terms of both local and global formability. The markedly enhanced global formability of the 3rd Gen 980 did not transfer to increased local formability as the fracture strains were comparable to or slightly lower than the DP980 steel.

## Figures and Tables

**Table 1:** Settings related to data/image acquisition and DIC for the 590R ^1^, DP980 ^2^, 3rd Gen 980 ^3^, 3rd Gen 1180 V1 ^4^, and 3rd Gen 1180 V2 ^5^. Note that the VSGL was computed from the product of the image resolution, steps size, and strain filter.

Test Method	Data/Image Acquisition Rate (Frames Per Second)	DIC Settings				
		Subset	Image Resolution (Pixel)	Step Size (Pixel)	Strain Filter	VSGL (Mm)
Tensile (0.001 s^−1^)	1.25 ^1^, 3.33 ^2^, 1.33 ^3^, 2 ^4^, 2 ^5^	29	0.073	3	5	~1
Tensile (1 s^−1^)	1000 ^1^, 2000 ^2,3,4,5^	13	0.026	3	7	~0.5
Tensile (100 s^−1^)	7 × 10^4 2^, 8.4 × 10^4 1,3,4,5^	13	0.051	2	5	~0.5
Tensile (1000 s^−1^)	9.52 × 10^5 1^, 1.25 × 10^6 2,3,4^, 1.11 × 10^6 5^	-	-	-	-	-
Interrupted tensile tests	2 ^1,4,5^, 0.5 ^3^	29	0.045	3	7	~1
Shear (quasi-static)	1 ^1,2,3,4,5^	37	0.011	7	5	~0.3
Marciniak tests	3–5 ^2^, 5–6 ^1,3,4,5^	35	0.057	2	9	~1
V-Bend	5 ^1^, 13 ^4^, 10 ^2,3,5^	29	0.041	3	5	~0.5
Miniature domes	7 ^1,3^, 7 ^2^, 4 ^4^, 5 ^5^	41	0.025	3	7	~0.5
Hole expansion	4 ^2^, 7 ^1,3,4,5^	-	-	-	-	-

**Table 2:** Mechanical properties obtained in quasi-static tensile tests in the TD. A 50 mm virtual extensometer was adopted for strain measurements and the uniform elongation was determined from the Considère Criterion.

Grade	Nominal Sheet Thickness (Mm)	Surface Condition	Yield Strength (Mpa)	Ultimate Tensile Strength (Mpa)	Yield-to-UTS Ratio	Uniform Elongation UE (%)	Total Elongation TE (%)
590R	1.4	uncoated	490 (±2)	671 (±1)	0.73	14.4 (±0.1)	23.7 (±0.4)
DP980	1.2	uncoated	735 (±2)	1065 (±3)	0.69	7.8 (±0.2)	13.7 (±0.5)
3rd Gen 980	1.4	uncoated	681 (±8)	1034 (±10)	0.66	18 (±0.5)	24.9 (±0.6)
DP1180	1.0	uncoated	843 (±0)	1216 (±8)	0.69	6.5 (±0.4)	11.5 (±0.2)
3rd Gen 1180 V1	1.4	electro-galvanized coating	950 (±12)	1251 (±8)	0.76	8.4 (±0.2)	14.1 (±0.6)
3rd Gen 1180 V2	1.4	electro-galvanized coating	1043 (±4)	1225 (±8)	0.85	10.7 (±0.4)	16.4 (±0.3)

**Table 3:** Calibrated coefficients for the MHS hardening model in the RD. Note that the data for the DP1180 was published in Butcher et al. [14], for the DP980 in Noder and Butcher [21], and for the 3rd Gen 1180 V1 and 3rd Gen 980 in Gutierrez et al. [12]. The conversion factor from RD to TD was obtained using the measured stress ratios.

Steel Grade	Modified Hockett-Sherby Model Parameters					
	*G* (MPa)	*H* (MPa)	*I*	*J*	*K* (MPa)	Plastic Strain at Uniform Elongation
590R	743.93	448.09	4.53	0.58	217.71	0.131
DP980	1072.87	604.90	11.54	0.50	327.25	0.069
3rd Gen 980	985.73	664.79	20.34	1.103	634.69	0.158
DP1180	1207.31	751.52	29.22	0.729	333.79	0.057
3rd Gen 1180 V1	1323.56	785.18	5.29	0.395	281.46	0.075
3rd Gen 1180 V2	1288.99	1063.62	28.40	1.260	355.90	0.093

**Table 4:** Calibrated coefficients for the empirical Yoshida model [29] to capture the chord modulus evolution with pre-strain. Note that interrupted tensile tests were not performed for the DP980 and DP1180. The DP980 and DP1180 data below was digitized from the study of Cobo et al. [31] and calibrated to Equation (6).

Steel Grade	*E*_0_ (GPa)	*E_S_* (GPa)	*ξ*
590R	206.6	162.6	31.9
DP980	209.9	174.7	129.3
3rd Gen 980	208.3	167.3	31.3
DP1180	209.6	181.7	346.7
3rd Gen 1180 V1	205.2	169.7	131.0
3rd Gen 1180 V2	209.0	176.1	145.9

**Table 5:** R-value and stress ratios of the studied 3rd Gen AHSS grades. Note that $\bar{R}$ refers to the normal anisotropy.

590R	w^pl^ [MJ/m^3^]	**σ_0/_σ_0_**	**σ_15/_σ_0_**	**σ_22.5/_σ_0_**	**σ_30/_σ_0_**	**σ_45/_σ_0_**	**σ_60/_σ_0_**	**σ_67.5/_σ_0_**	**σ_75/_σ_0_**	**σ_90/_σ_0_**	**τ_0/_σ_0_**	**τ_22.5/_σ_0_**	**τ_45/_σ_0_**
	84.4	1.000 (±0.003)	-	0.996 (±0.004)	-	0.995 (±0.003)	-	1.006 (±0.005)	-	1.019 (±0.002)	-	-	0.580
	Range plastic strain (R-value)	***R*_0_**	***R*_15_**	***R*_22.5_**	***R*_30_**	***R*_45_**	***R*_60_**	***R*_67.5_**	***R*_75_**	***R*_90_**	***R*_B_**	$\bar{R}$	
	0.02–0.10	0.67 (±0.01)	-	0.82 (±0.01)	-	1.08 (±0.01)	-	1.00 (±0.01)	-	0.90 (±0.02)	-	0.93 (±0.01)	
DP980	w^pl^ [MJ/m^3^]	**σ_0/_σ_0_**	**σ_15/_σ_0_**	**σ_22.5/_σ_0_**	**σ_30/_σ_0_**	**σ_45/_σ_0_**	**σ_60/_σ_0_**	**σ_67.5/_σ_0_**	**σ_75/_σ_0_**	**σ_90/_σ_0_**	**τ_0/_σ_0_**	**τ_22.5/_σ_0_**	**τ_45/_σ_0_**
	70.00	1.000 (±0.013)	1.012 (±0.007)	-	0.995 (±0.003)	0.988 (±0.005)	1.009 (±0.004)	-	1.018 (±0.004)	1.018 (±0.003)	0.591	0.581	0.608
	Range plastic strain (R-value)	***R*_0_**	***R*_15_**		***R*_30_**	***R*_45_**	***R*_60_**	***R*_67.5_**	***R*_75_**	***R*_90_**	***R*_B_**	$\bar{R}$	
	0.01–0.05	0.78 (±0.02)	0.79 (±0.02)	-	0.86 (±0.03)	1.03 (±0.01)	0.96 (±0.01)	-	0.87 (±0.03)	0.95 (±0.01)	0.84 (±0.06)	0.95 (±0.01)	
3rd Gen 980	w^pl^ [MJ/m^3^]	**σ_0/_σ_0_**	**σ_15/_σ_0_**	**σ_22.5/_σ_0_**	**σ_30/_σ_0_**	**σ_45/_σ_0_**	**σ_60/_σ_0_**	**σ_67.5/_σ_0_**	**σ_75/_σ_0_**	**σ_90/_σ_0_**	**τ_0/_σ_0_**	**τ_22.5/_σ_0_**	**τ_45/_σ_0_**
	164.7	1.000 (±0.013)	-	0.981 (±0.001)	-	0.971 (±0.006)	-	0.979 (±0.003)	-	0.998 (±0.008)	-	-	0.585
	Range plastic strain (R-value)	***R*_0_**	***R*_15_**	***R*_22.5_**	***R*_30_**	***R*_45_**	***R*_60_**	***R*_67.5_**	***R*_75_**	***R*_90_**	***R*_B_**	$\bar{R}$	
	0.05–0.15	0.86 (±0.01)	-	0.86 (±0.01)	-	0.93 (±0.01)	-	0.91 (±0.01)	-	0.90 (±0.00)	1.00 (±0.05)	0.90 (±0.00)	
DP1180	w^pl^ [MJ/m^3^]	**σ_0/_σ_0_**	**σ_15/_σ_0_**	**σ_22.5/_σ_0_**	**σ_30/_σ_0_**	**σ_45/_σ_0_**	**σ_60/_σ_0_**	**σ_67.5/_σ_0_**	**σ_75/_σ_0_**	**σ_90/_σ_0_**	**τ_0/_σ_0_**	**τ_22.5/_σ_0_**	**τ_45/_σ_0_**
	61.11	1.000 (±0.006)	0.995 (±0.003)	-	0.996 (±0.003)	1.004 (±0.007)	1.008 (±0.008)	-	1.013 (±0.003)	1.025 (±0.007)	0.600	0.600	0.612
	Range plastic strain (R-value)	***R*_0_**	***R*** **_15_**		***R*_30_**	***R*_45_**	***R*_60_**	***R*_67.5_**	***R*_75_**	***R*_90_**	***R*_B_**	$\bar{R}$	
	0.01–0.05	0.82 (±0.01)	0.84 (±0.01)	-	0.90 (±0.01)	0.95 (±0.01)	0.98 (±0.01)	-	1.00 (±0.00)	0.98 (±0.01)	0.94 (±0.03)	0.93 (±0.01)	
3rd Gen 1180 V1	w^pl^ [MJ/m^3^]	**σ_0/_σ_0_**	**σ_15/_σ_0_**	**σ_22.5/_σ_0_**	**σ_30/_σ_0_**	**σ_45/_σ_0_**	**σ_60/_σ_0_**	**σ_67.5/_σ_0_**	**σ_75/_σ_0_**	**σ_90/_σ_0_**	**τ_0/_σ_0_**	**τ_22.5/_σ_0_**	**τ_45/_σ_0_**
	88.4	1.000 (±0.015)	-	0.996 (±0.010)	-	1.001 (±0.004)	-	1.010 (±0.007)	-	1.022 (±0.006)	-	-	0.618
	Range plastic strain (R-value)	***R*_0_**	***R*_15_**	***R*_22.5_**	***R*** **_30_**	***R*** **_45_**	***R*_60_**	***R*** **_67.5_**	***R*** **_75_**	***R*_90_**	***R*_B_**	$\bar{R}$	
	0.03–0.08	0.76 (±0.01)	-	0.83 (±0.00)	-	0.93 (±0.00)	-	0.90 (±0.02)	-	0.90 (±0.01)	0.92 (±0.03)	0.88 (±0.01)	
3rd Gen 1180 V2	w^pl^ [MJ/m^3^]	**σ_0/_σ_0_**	**σ_15/_σ_0_**	**σ_22.5/_σ_0_**	**σ_30/_σ_0_**	**σ_45/_σ_0_**	**σ_60/_σ_0_**	**σ_67.5/_σ_0_**	**σ_75/_σ_0_**	**σ_90/_σ_0_**	**τ_0/_σ_0_**	**τ_22.5/_σ_0_**	**τ_45/_σ_0_**
	107.8	1.000 (±0.002)	-	0.993 (±0.001)	-	0.989 (±0.003)	-	0.993 (±0.006)	-	0.998 (±0.006)	-	-	0.557
	Range plastic strain (R-value)	***R*_0_**	***R*_15_**	***R*_22.5_**	***R*_30_**	***R*_45_**	***R*_60_**	***R*_67.5_**	***R*_75_**	***R*_90_**	***R*_B_**	$\bar{R}$	
	0.03–0.08	0.89 (±0.00)	-	0.91 (±0.00)	-	0.94 (±0.01)	-	0.93 (±0.00)	-	0.89 (±0.00)	-	0.92 (±0.00)	

**Table 6:** Calibration coefficients of the Barlat Yld2000 yield function. Note that the data for the DP1180 was retrieved from the study of Butcher et al. [14] and the 3rd Gen 980 and 1180 V1 from Gutierrez et al. [12].

Steel Grade	Barlat Yld2000 Calibration Coefficients								
	α_1_	α_2_	α_3_	α_4_	α_5_	α_6_	α_7_	α_8_	*m*
590R	0.792	1.119	1.105	0.989	1.0143	0.842	0.999	0.995	4.8
DP980	0.955	0.992	0.989	0.987	1.026	1.016	1.002	1.037	4.8
3rd Gen 980	0.970	1.005	1.000	1.007	1.011	0.992	1.015	1.077	6
DP1180	0.966	0.982	0.998	0.979	1.000	0.927	0.990	1.036	6
3rd Gen 1180 V1	0.969	0.946	0.978	0.998	1.016	0.964	0.993	1.066	4.7
3rd Gen 1180 V2	0.995	0.987	0.976	1.004	1.009	1.015	1.004	1.037	5.6

**Table 7:** Calibrated coefficients for the rate-dependent constitutive model in Equations (7) and (8). Note that a reference strain rate of 0.001 s^−1^ was adopted.

	Model Parameters: Quadratic Johnson Cook or Modified Cowper–Symonds			
Steel Grade	*X*	*Y*	*C*	*p*
590R	4.5 × 10^−3^	4.66 × 10^−^^4^	-	-
DP980	-	-	49.31	0.521
3rd Gen 980	3.38 × 10^−3^	4.55 × 10^−^^4^	-	-
3rd Gen 1180 V1	−5.00 × 10^−4^	9.27 × 10^−^^4^	-	-
3rd Gen 1180 V2	-	-	55.92	0.485

**Table 8:** Surface roughness and coefficient of friction for different steel grades. Note that the friction coefficient was obtained using the lubricant CommDraw^TM^ 220. The numbers in brackets correspond to the standard deviation.

Process Parameter	590R	3rd Gen 980	3rd Gen 1180 V1	3rd Gen 1180 V2
COF	0.11 (±0.01)	0.13 (±0.01)	0.19 (±0.01)	0.15 (±0.01)
Mean surface roughness R_a_ (µm)	1.02 (±0.1)	1.07 (±0.07)	1.08 (±0.08)	0.58 (±0.05)
Total height of roughness R_t_ (µm)	8.28 (±1.61)	7.97 (±0.90)	9.16 (±1.97)	4.97 (±1.31)

**Table 9:** Calibration coefficients for the GDP fracture *loci* for the studied steel grades.

Steel Grades	*a*	*b*	*c*	*β*
590R	0.6553	−0.4043	0.2729	1.2691
DP980	0.3625	−0.4864	0.8645	1.6166
3rd Gen 980	0.4308	−0.6587	1.0323	1.2926
DP1180	0.2162	−1.5997	3.8744	3.1733
3rd Gen 1180 V1	0.2166	0.2858	3.6450	3.2050
3rd Gen 1180 V2	0.3646	−0.7119	0.9778	1.5229

## Data Availability

Not applicable.

## Appendix A

**Table A1:** Forming limit strains obtained in Marciniak tests for the 590R.

Repeat	Geometry #2		Geometry #3		Geometry #4		Geometry #5	
	ε_1_	ε_2_	ε_1_	ε_2_	ε_1_	ε_2_	ε_1_	ε_2_
R1	0.338	−0.101	0.256	−0.057	0.193	−0.011	0.303	0.033
R2	0.336	−0.098	0.255	−0.056	0.190	−0.012	0.262	0.024
R3	0.338	−0.098	0.255	−0.062	0.186	−0.008	0.254	0.021
R4	0.335	−0.100	0.259	−0.059	0.205	−0.015		
R5	0.336	−0.105						
Average	0.336	−0.100	0.256	−0.058	0.193	−0.012	0.273	0.026
Std	0.001	0.002	0.002	0.003	0.003	0.002	0.026	0.007

**Table A2:** Forming limit strains obtained in Marciniak tests for the 3rd Gen 1180 V2.

Repeat	Geometry #1		Geometry #2		Geometry #3		Geometry #4		Geometry #5		Geometry #6	
	ε_1_	ε_2_	ε_1_	ε_2_	ε_1_	ε_2_	ε_1_	ε_2_	ε_1_	ε_2_	ε_1_	ε_2_
R1	0.238	−0.104	0.206	−0.075	0.171	−0.043	0.099	0.000	0.162	0.036	0.327	0.287
R2	0.205	−0.093	0.215	−0.079	0.176	−0.043	0.098	0.000	0.143	0.028	0.287	0.274
R3	0.206	−0.089	0.209	−0.071	0.173	−0.037	0.099	−0.001	0.140	0.032	0.344	0.316
R4	0.206	−0.092	0.199	−0.071	0.177	−0.037	0.122	0.002	0.164	0.035	0.300	0.279
R5			0.199	−0.074								
Average	0.214	−0.095	0.206	−0.074	0.174	−0.040	0.105	0.000	0.152	0.033	0.315	0.289
Std	0.016	0.007	0.007	0.004	0.003	0.004	0.001	0.000	0.013	0.003	0.026	0.019

**Table A3:** Summary of fracture strains for the 590R AHSS. Note that the von Mises effective strain corresponds to the absolute strain assuming a linear strain path. The Barlat Yld2000 equivalent strain corresponds to the *accumulated* work-conjugate equivalent strain integrated along the measured strain path and accounts for material anisotropy.

Stress State	590R				
	ε_1_	ε_2_	ε_eff VM_	ε_eq Yld2000_	Triaxiality
Shear	0.787 (±0.028)	−0.813 (±0.020)	1.006 (±0.030)	0.906 (±0.025)	−0.027 (±0.014)
UT	1.010 (±0.069)	−0.473 (±0.031)	1.010 (±0.066)	0.997 (±0.065)	0.335 (±0.000)
PST	0.767 (±0.005)	0.001 (±0.001)	0.890 (±0.010)	0.856 (±0.005)	0.584 (±0.001)
EB	0.773 (±0.013)	0.680 (±0.040)	1.460 (±0.050)	1.461 (±0.034)	0.666 (±0.000)

**Table A4:** Summary of fracture strains for the DP980 AHSS. Note that the von Mises effective strain corresponds to the absolute strain assuming a linear strain path. The Barlat Yld2000 equivalent strain corresponds to the *accumulated* work-conjugate equivalent strain integrated along the measured strain path and accounts for material anisotropy.

Stress State	DP980				
	ε_1_	ε_2_	ε_eff VM_	ε_eq Yld2000_	Triaxiality
Shear	0.608 (±0.02)	−0.605 (±0.019)	0.750 (±0.030)	0.712 (±0.011)	0.011 (±0.009)
UT	0.850 (±0.050)	−0.415 (±0.022)	0.850 (±0.050)	0.867 (±0.047)	0.332 (±0.000)
PST	0.470 (±0.030)	0.000 (±0.000)	0.540 (±0.040)	0.541 (±0.038)	0.573 (±0.001)
EB	0.604 (±0.009)	0.560 (±0.020)	1.160 (±0.020)	1.173 (±0.010)	0.666 (±0.000)

**Table A5:** Summary of fracture strains for the 3rd Gen 980 AHSS. Note that the von Mises effective strain corresponds to the absolute strain assuming a linear strain path. The Barlat Yld2000 equivalent strain corresponds to the *accumulated* work-conjugate equivalent strain integrated along the measured strain path and accounts for material anisotropy.

Stress State	3rd Gen 980				
	ε_1_	ε_2_	ε_eff VM_	ε_eq Yld2000_	Triaxiality
Shear	0.599 (±0.006)	−0.599 (±0.007)	0.734 (±0.010)	0.677 (±0.008)	−0.013 (±0.004)
UT	0.638 (±0.034)	−0.302 (±0.016)	0.640 (±0.030)	0.636 (±0.034)	0.333 (±0.000)
PST	0.429 (±0.001)	0.001 (±0.001)	0.500 (±0.020)	0.475 (±0.019)	0.581 (±0.002)
EB	0.498 (±0.017)	0.448 (±0.063)	0.950 (±0.050)	0.941 (±0.078)	0.666 (±0.000)

**Table A6:** Summary of fracture strains for the DP1180 AHSS. Note that the von Mises effective strain corresponds to the absolute strain assuming a linear strain path. The Barlat Yld2000 equivalent strain corresponds to the *accumulated* work-conjugate equivalent strain integrated along the measured strain path and accounts for material anisotropy.

Stress State	DP1180				
	ε_1_	ε_2_	ε_eff VM_	ε_eq Yld2000_	Triaxiality
Shear	0.587 (±0.020)	−0.567 (±0.008)	0.720 (±0.030)	0.661 (±0.017)	0.049 (±0.019)
UT	0.635 (±0.025)	−0.314 (±0.012)	0.640 (±0.030)	0.651 (±0.023)	0.334 (±0.000)
PST	0.340 (±0.009)	0.001 (±0.001)	0.390 (±0.010)	0.380 (±0.011)	0.573 (±0.002)
EB	0.502 (±0.013)	0.449 (±0.009)	0.950 (±0.050)	0.969 (±0.015)	0.666 (±0.000)

**Table A7:** Summary of fracture strains for the 3rd Gen 1180 V1 AHSS. Note that the von Mises effective strain corresponds to the absolute strain assuming a linear strain path. The Barlat Yld2000 equivalent strain corresponds to the *accumulated* work-conjugate equivalent strain integrated along the measured strain path and accounts for material anisotropy.

Stress State	3rd Gen 1180 V1				
	ε_1_	ε_2_	ε_eff VM_	ε_eq Yld2000_	Triaxiality
Shear	0.560 (±0.000)	−0.568 (±0.007)	0.676 (±0.010)	0.668 (±0.008)	0.007 (±0.005)
UT	0.680 (±0.030)	−0.322 (±0.016)	0.680(±0.030)	0.693 (±0.034)	0.320 (±0.000)
PST	0.279 (±0.019)	0.001 (±0.001)	0.320 (±0.020)	0.319 (±0.022)	0.580 (±0.002)
EB	0.391 (±0.019)	0.291 (±0.079)	0.700 (±0.050)	0.687 (±0.090)	0.664 (±0.003)

**Table A8:** Summary of fracture strains for the 3rd Gen 1180 V2 AHSS. Note that the von Mises effective strain corresponds to the absolute strain assuming a linear strain path. The Barlat Yld2000 equivalent strain corresponds to the *accumulated* work-conjugate equivalent strain integrated along the measured strain path and accounts for material anisotropy.

Stress State	3rd Gen 1180 V2				
	ε_1_	ε_2_	ε_eff VM_	ε_eq Yld2000_	Triaxiality
Shear	0.595 (±0.008)	−0.604 (±0.004)	0.728 (±0.012)	0.675 (±0.005)	−0.009 (±0.016)
UT	0.772 (±0.053)	−0.363 (±0.025)	0.770 (±0.050)	0.770 (±0.053)	0.337 (±0.000)
PST	0.459 (±0.022)	0.001 (±0.001)	0.530 (±0.010)	0.512 (±0.025)	0.585 (±0.001)
EB	0.622 (±0.047)	0.586 (±0.041)	1.160 (±0.080)	1.203 (±0.086)	0.667 (±0.000)

## Appendix B

The partial derivatives of the Modified Maximum Force Criterion of Hora et al. [47] in Equation (14) can be further expanded. The partial derivative of the first principal stress with respect to the first principal strain is

(A1) $$\begin{array}{c} \frac{\partial \sigma_{1}}{\partial \varepsilon_{1}}=\frac{\partial \sigma_{1}}{\partial \sigma_{eq}}\frac{\partial \sigma_{eq}}{\partial \bar{\sigma }}\frac{\partial \bar{\sigma }}{\partial \varepsilon_{eq}}\frac{\partial \varepsilon_{eq}}{\partial \varepsilon_{1}}=hk^{2}\left(1+\alpha \rho \right)\\ \mathrm{Von}\;\mathrm{Mises}:\;\frac{\partial \sigma_{1}}{\partial \varepsilon_{1}}=\frac{h}{\left(1-\alpha +\alpha^{2}\right)}\left(1+\alpha \rho \right) \end{array}$$

where $h=\frac{\partial \bar{\sigma }}{\partial \varepsilon_{eq}}$ refers to the material hardening rate and $k\left(\alpha \right)=\frac{\sigma_{1}}{\sigma_{eq}}$ to the ratio of the principal stress to the equivalent stress. The derivative of the principal stress with respect to the principal in-plane strain ratio, ρ, is

(A2) $$\frac{\partial \sigma_{1}}{\partial \rho }=\frac{\partial \sigma_{1}}{\partial \alpha }\frac{\partial \alpha }{\partial \rho }=\left(\frac{\partial k}{\partial \alpha }\sigma_{eq}\right)\frac{\partial \alpha }{\partial \rho }=\frac{\sigma_{eq}\left(\partial k/\partial \alpha \right)}{\left(\partial \rho /\partial \alpha \right)}$$

(A3) $$\frac{\partial k}{\partial \alpha }=\frac{\partial k}{\partial \sigma_{eq}}\frac{\partial \sigma_{eq}}{\partial \sigma_{2}}\frac{\partial \sigma_{2}}{\partial \alpha }=-k^{2}\frac{\partial \sigma_{eq}}{\partial \sigma_{2}}\to \;\mathrm{Von}\;\mathrm{Mises}:\;\frac{\partial k}{\partial \alpha }=\frac{\left(1-2\alpha \right)}{2{\left(1-\alpha +\alpha^{2}\right)}^{\frac{3}{2}}}$$

(A4) $$\frac{\partial \rho }{\partial \alpha }=\sigma_{1}^{}{\left(\frac{\partial \sigma_{eq}}{\partial \sigma_{1}^{}}\right)}^{-1}\left(\frac{\partial^{2}\sigma_{eq}}{\partial \sigma_{2}^{2}}-\rho \frac{\partial^{2}\sigma_{eq}}{\partial \sigma_{1}^{}\partial \sigma_{2}^{}}\right)\to \;\mathrm{Von}\;\mathrm{Mises}:\;\frac{\partial \rho }{\partial \alpha }=\frac{3}{{\left(\alpha -2\right)}^{2}}$$

## Appendix C

Calibration of the fracture locus for this study was performed in a deterministic manner such that a functional form for the parameters *b*, *c*, and *β* was derived with an explicit dependence upon the Hosford yield exponent, *a*, which was then solved numerically using the Newton–Raphson method. Note that the deterministic identification of the calibration parameters was not possible for the 3rd Gen 1180 V1, which was calibrated utilizing a best fit approach.

For this purpose, the GDP function in Equation (23) was simplified for the respective stress states. For uniaxial tension $\left(\sigma_{1}=1,\;\sigma_{2}=\sigma_{3}=0,\;{\bar{\theta }}_{L}=1,\;T=\frac{1}{3}\right)$ the GDP function simplifies to

(A5) $$g_{GDP}^{UT}\left[\sigma_{ij}/\sigma_{eq}^{von\;Mises}\right]=1+c$$

For equi-biaxial tension $\left(\sigma_{1}=1,\;\sigma_{2}=\sigma_{1}=1,\;\sigma_{3}=0,\;{\bar{\theta }}_{L}=-1,\;T=\frac{2}{3}\right)$, the GDP function reduces to

(A6) $$g_{GDP}^{EB}\left[\sigma_{ij}/\sigma_{eq}^{von\;Mises}\right]=1+c+b$$

The GDP function takes on the following form for simple shear loading $\left(\sigma_{1}=1,\;\sigma_{2}=0,\;\sigma_{3}=-\sigma_{1}=-1,\;{\bar{\theta }}_{L}=0,\;T=0\right)$

(A7) $$g_{GDP}^{SS}\left[\sigma_{ij}/\sigma_{eq}^{von\;Mises}\right]={\left(\frac{1}{2}\left({\left|{\left\{f_{1}-f\right\}}_{2}\right|}^{a}+{\left|{\left\{f_{2}-f\right\}}_{3}\right|}^{a}+{\left|{\left\{f_{1}-f\right\}}_{3}\right|}^{a}\right)\right)}^{\frac{1}{a}}=\sigma_{Hosford}^{eq,SS}$$

and for plane strain tension $\left(\sigma_{1}=1,\;\sigma_{2}=\frac{1}{2},\;\sigma_{3}=0,\;{\bar{\theta }}_{L}=0,\;T=\frac{1}{\sqrt{3}}\right)$, the GDP function yields

(A8) $$\begin{array}{cc} g_{GDP}^{PST}\left[\sigma_{ij}/\sigma_{eq}^{von\;Mises}\right] & ={\left(\frac{1}{2}\left({\left|\left\{f_{1}-f_{2}\right\}\right|}^{a}+{\left|\left\{f_{2}-f_{3}\right\}\right|}^{a}+{\left|\left\{f_{1}-f_{3}\right\}\right|}^{a}\right)\right)}^{\frac{1}{a}}+\frac{2c}{\sqrt{3}}+\frac{b}{\sqrt{3}}\\ & =\sigma_{Hosford}^{eq,SS}+\frac{2c}{\sqrt{3}}+\frac{b}{\sqrt{3}} \end{array}$$

Since the equivalent fracture strains are known, Equation (28) can be utilized to derive a functional form of the material parameters in dependence of the Hosford yield exponent. Substitution of the equivalent fracture strains and adoption of Equations (A5)–(A7) yields

(A9) $$c=\frac{\beta^{\prime }}{\varepsilon_{eq}^{UT}}-1,\;b=\frac{\beta^{\prime }}{\varepsilon_{eq}^{EB}}-1-c,\;\beta^{\prime }=\varepsilon_{eq}^{SS}\sigma_{Hosford}^{eq,SS}$$

which is then substituted into Equation (A8) for plane strain tension to arrive at the objective function utilized in the Newton–Raphson method to solve for the yield function exponent in the Hosford equivalent stress

(A10) $$\varepsilon_{eq}^{PST}\left(\sigma_{Hosford}^{eq,SS}+\frac{2c}{\sqrt{3}}+\frac{b}{\sqrt{3}}\right)-\beta^{\prime }=0\;$$
